# Room-Temperature Electrocatalytic Dehydrogenation and Partial Fragmentation of *n*‑Alkanes

**DOI:** 10.1021/jacs.5c12806

**Published:** 2025-09-22

**Authors:** Gong Zhang, Lee Fuller, Christine Lucky, Alexander J. Zielinski, Dongfang Cheng, Enner Mendoza, Philippe Sautet, Marcel Schreier

**Affiliations:** ‡ Department of Chemical and Biological Engineering, University of Wisconsin−Madison, Madison, Wisconsin 53706, United States; † Department of Chemistry, University of Wisconsin−Madison, Madison, Wisconsin 53706, United States; ⊥ Department of Chemical and Biomolecular Engineering, University of California, Los Angeles, California 90095, United States; § Department of Chemistry and Biochemistry, University of California, Los Angeles, California 90095, United States; # California NanoSystems Institute, Los Angeles, California 90095, United States

## Abstract

Light alkenes are central building blocks in the chemical industry, yet their production from alkanes requires energy-intensive processes that generate substantial CO_2_ emissions and suffer from catalyst deactivation and overoxidation. In this work, we demonstrate that alkane dehydrogenation can be accomplished at ambient temperature and pressure by modulating the voltage applied to an electrocatalyst surface. Voltage manipulation provides real-time control over the adsorption and dehydrogenation of alkanes, as well as the potential-driven desorption of dehydrogenated adsorbates. By using a newly developed sensitive gas chromatographic (GC) analysis method, we were able to quantify the formation of 1-butene from *n*-butane, as well as the formation of a distribution of shorter chain alkanes and alkenes. We show that the product distribution depends on the potential applied during adsorption and is sensitive to both catalyst identity and electrolyte composition. Palladium suppresses C–C bond cleavage relative to platinum, and replacing protons with sodium cations increases 1-butene selectivity by promoting desorption over hydrogenation. To rationalize the observed product distribution, we propose a reaction mechanism supported by grand canonical density functional theory calculations. Together, these results reveal a new pathway for alkane dehydrogenation under ambient conditions and establish time-programmed electrochemical control as a promising tool for manipulating surface-catalyzed transformations of *n*-alkanes.

## Graphical abstract

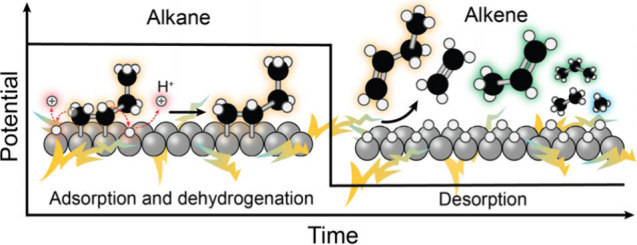

Light alkenes are vital feedstocks in the production of synthetic fibers, rubber, plastics, and a wide range of other industrial products.^1^ Traditionally, alkene production relies on naphtha steam cracking.^2^ However, rising availability of inexpensive shale gas has sparked interest in the direct dehydrogenation of short-chain alkanes.^3^ To meet this demand, several alkane dehydrogenation processes have been developed, such as the Catadiene, Catofin and Oleflex process, among others.^4^ Yet, the endothermic nature of alkane dehydrogenation dictates that these processes must operate at high temperatures, resulting in substantial carbon emissions.^4,5^ High-temperature dehydrogenation processes also suffer from catalyst deactivation through coke deposition.^6,7^ Attempts to lower the operating temperature by introducing oxidizers (such as O_2_) to accept the released hydrogen continue to suffer from poor selectivity due to product overoxidation.^8,9^

An elegant route for displacing the reaction equilibrium at lower temperature would be the use of electrochemistry. At its heart, dehydrogenation can be seen as a proton-coupled electron transfer reaction, wherein two electrons and protons are removed from an alkane molecule to form a C=C double bond (Scheme 1a). The equilibrium of this reaction can be freely controlled by the potential applied to a catalyst surface, which would – in principle – allow for dehydrogenation to take place at room temperature.

**Scheme 1:**
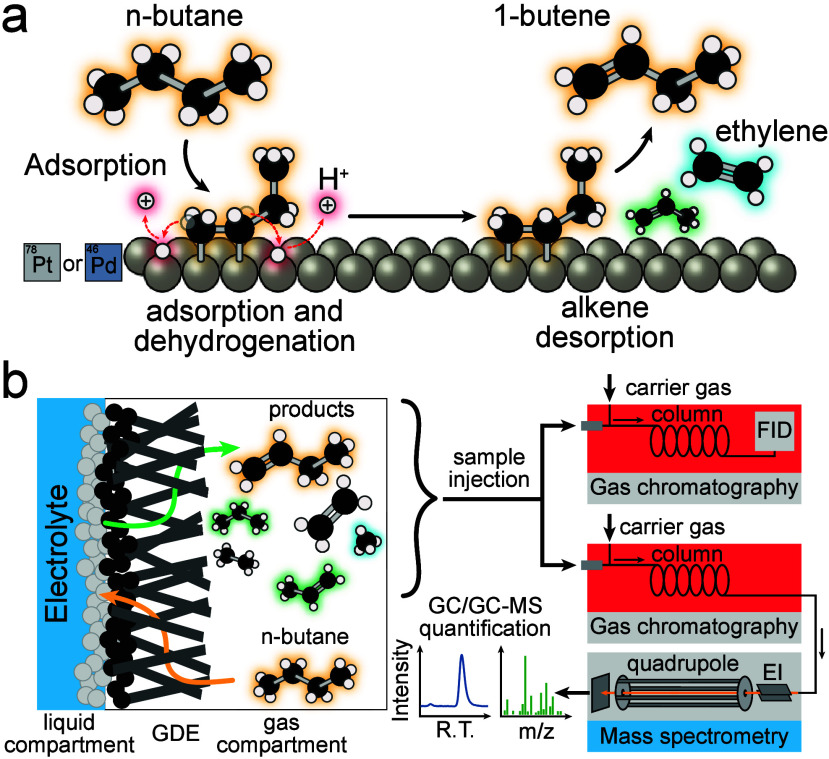
Electrochemical Alkane Dehydrogenation: (a) Illustration of a Possible Electrocatalytic Dehydrogenation and Partial Fragmentation Pathway for *n*-Butane on Pt and Pd Electrodes;^a^ (b) Schematic of the Gas Diffusion Electrode Structure and the Product Analysis System Used in This Work^b^

a The reactions are facilitated by modulation of the surface hydrogen-proton equilibrium, which promotes the dissociative adsorption of alkanes, leading to the formation of dehydrogenated intermediates. These intermediates can subsequently undergo desorption as alkenes, driven by controlled potential steps. The illustrations are not to scale.
b EI, electron ionization source. R.T., retention time.

Recent reports have demonstrated propane dehydrogenation near ambient conditions using stoichiometric cuprous oxide as a sacrificial hydrogen acceptor in acidic solution and in the presence of molecular oxygen.^5,10,11^ A key drawback of this method is the continuous dissolution of the active oxide layer in the acidic solution. To counteract this instability, the same authors more recently used an electrochemical cell, where an oxidative potential at the anode formed the copper oxide for C–H dissociation in the presence of oxygen, while the cathode redeposited the dissolved Cu^2+^ ions, with periodic reversal of potential.

Beyond generating sacrificial hydrogen acceptors, however, the ability to adapt the electrochemical potential in a time-dependent manner holds the promise of providing unprecedented control over the processes involved in *catalytic* dehydrogenation reactions on nonsacrificial catalyst surfaces. Recent experimental work by our group has demonstrated that time-dependent application of a series of potentials to electrocatalyst surfaces can be used to independently control individual steps in electrocatalytic reactions.^12–16^ This finding, which was inspired by early work on alkane fuel cells and has recently been confirmed by others,^17,18^ opens avenues to use renewable electricity to accomplish reactions at room temperature that traditionally require high-temperatures to take place. For example, we demonstrated the room-temperature fragmentation (C–C cleavage) of *n*-alkanes by adsorbing alkanes to platinum (Pt) electrodes in the vicinity of the potential of zero charge (PZC),^19–23^ followed by the application of more oxidative potentials to enable C–C bond cleavage. Subsequent application of cathodic potentials allowed us to initiate the desorption of fragmented products.^24^ The use of these potential profiles allowed each step of the reaction to proceed under locally optimized conditions.^16,25^

We hypothesized that our time-controlled potential methodologies would allow us to accomplish *n*-alkane dehydrogenation at electrocatalyst surfaces under ambient conditions. Critical to achieving this goal is controlling the population of hydrogen atoms (*H) on the catalyst surface. At oxidative potentials, the hydrogen generated through the dissociative adsorption (C–H dissociation and formation of a C–metal bond) of alkanes is removed from the electrode via oxidation to protons.^26,27^ Thereby, the potential can control the adsorption equilibrium of alkanes, and the propensity of the resulting fragments to dehydrogenate. Indeed, electrochemical alkane adsorption has been suggested to produce dehydrogenated hydrocarbon intermediates at electrode surfaces.^15,28–33^ We therefore theorized that desorbing these intermediates in their dehydrogenated form would open an avenue to the room-temperature electrochemical dehydrogenation of alkanes on catalyst surfaces.

Realizing this route to alkane dehydrogenation requires overcoming several technical challenges. Among these is ensuring the accurate analysis of the generated products. Several studies of our group and others used electrochemical mass spectrometry (EC-MS) for product detection. This method has limitations, however, as mixtures of alkanes and alkenes form fragments of similar mass to charge ratio (*m*/*z*) during the ionization process.^34^ Moreover, recent studies have shown that when the MS intensity at a given *m*/*z* increases during a reaction, this increase may not only result from ionization or fragmentation of the expected product, but also from fragments formed via chemical ionization occurring in the electron ionization chamber.^35^ This limits the sensitivity toward products that feature similar types of fragments, which makes it challenging to analyze dehydrogenation products in the presence of mixtures of alkanes.

To overcome these limitations, we developed novel methods that allowed us to characterize the individual steps of dehydrogenation experiments using monolayer-sensitive gas chromatography (GC). To achieve this, we carried out dehydrogenation experiments under real-time potential control on gas diffusion electrodes (GDEs, Scheme 1b). GDEs create a three-phase boundary between a liquid electrolyte layer, the electrocatalyst, and a gas phase. This results in high rates of mass transport between the catalyst surface and the gas phase, and offers sufficient catalyst surface area to allow for GC detection of monolayers of desorbed products.

Herein, using *n*-butane as a model compound, we show that the electrocatalytic dehydrogenation of *n*-alkanes can be realized. We also show that dehydrogenation coexists with alkane fragmentation processes that are modulated by the electrolyte composition and lead to the release of a mixture of shorter-chain dehydrogenated compounds.

We started our experiments by using GC to explore the products resulting from the adsorption of *n*-butane on Pt at a series of adsorption potentials, followed by reductive desorption at a fixed potential (0.05 V vs the standard hydrogen electrode, SHE), as shown in Figure 1a. This desorption potential was chosen to lie within the hydrogen underpotential deposition (H_UPD_) region (Figure S1).^36,37^ Our GDEs were prepared by sputter coating a ∼100 nm thick catalyst layer onto commercial hydrophobic carbon paper, ensuring excellent electrical conductivity. The catalyst layer was in direct contact with the electrolyte, while the hydrophobic backing layer contacted the gas phase (Figure S2). We used a three-electrode setup, employing a leakless Ag/AgCl reference electrode, and a Pt foil as the counter electrode. All experiments were carried out in aqueous 1 M perchloric acid (HClO_4_, pH = 0) electrolyte, unless otherwise specified.

**Figure 1:**
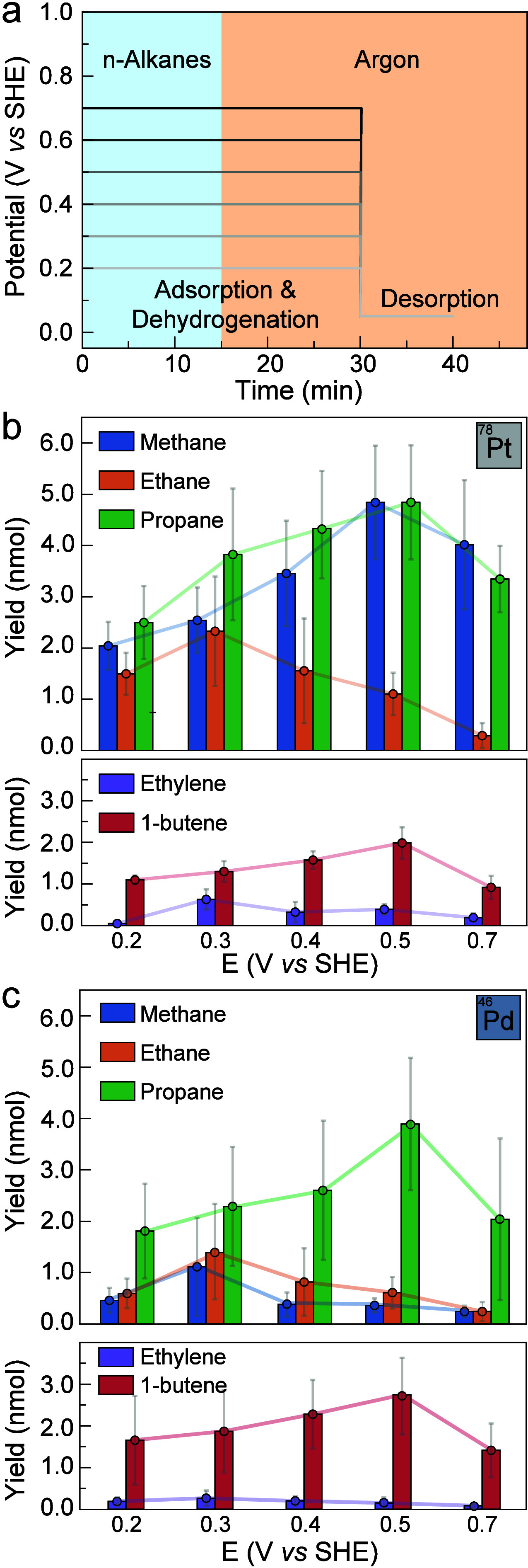
GC results of electrochemical *n*-butane dehydrogenation and fragmentation using different GDEs. (a) Illustration of the applied potential program. (b) Product distribution observed from Pt GDEs. (c) Product distribution observed from Pd GDEs. Unconverted *n*-butane was not quantified. Three independent experiments were conducted using three GDE electrodes. Error bars represent standard deviations from triplicate measurements, with primary variability arising from slight differences in GDE catalyst loading.

During the desorption step, the gaseous products were allowed to accumulate in the gas compartment of the cell and were subsequently withdrawn using a syringe (Figure S3). The gas composition was then analyzed using GC. Representative chromatograms are shown in Figures S4 and S5 and full experimental details are provided in the Supporting Information.

Analysis of the gas after desorption revealed the release of unconverted *n*-butane along with cracking products such as methane, ethane, and propane. We had not previously observed the formation of a distribution of C_3_, C_2_, and C_1_ fragmentation products from the release of adsorbed alkanes, likely due to the difficulty in distinguishing different alkanes with overlapping *m*/*z* fragments in mass spectrometry. This is overcome by the higher sensitivity and specificity of our new GC method. In addition to the fragmentation products, we also observed the formation of 1-butene, indicating that alkanes can be desorbed in the form of alkenes (Figure 1b). We also observed the production of small amounts of ethylene and trace quantities of propene (Figure 2), corresponding to dehydrogenated fragmentation products. Overall, our data indicates that alkanes can be successfully dehydrogenated at room temperature by leveraging time-dependent changes in potential.

**Figure 2:**
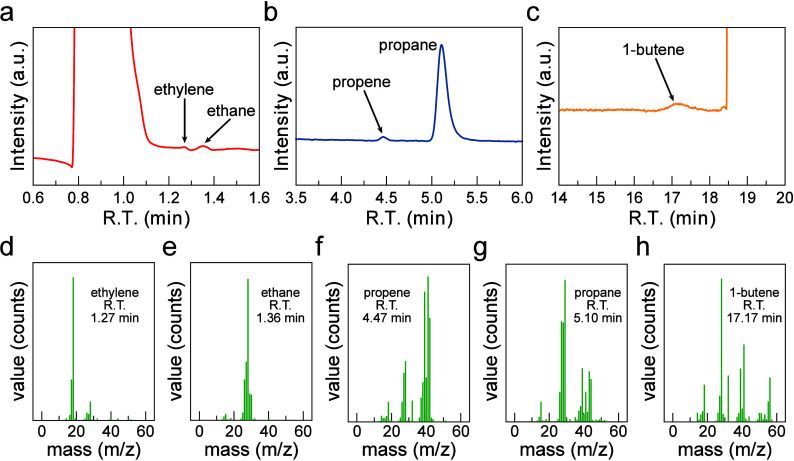
Results of GC-MS experiments of *n*-butane dehydrogenation on a Pt GDE. (a) Total ion chromatography (TIC) of ethylene and ethane peaks. (b) TIC plot of propene and propane peaks. (c) TIC plot of the 1-butene peak. GC-MS signals of (d) ethylene, (e) ethane, (f) propene, (g) propane, (h) 1-butene corresponding to different retention times. The adsorption potential for these measurements was 0.5 V vs SHE. R.T., retention time. The complete TIC chromatogram can be found in Figure S6.

To validate the product identification obtained through GC, we performed GC-MS analyses of the products. These analyses showed the appearance of mass fragments corresponding to the expected dehydrogenation and fragmentation products (Figure 2 and Figure S6–9). It is worth noting that trace amounts of propene were also detected using GC-MS. However, due to its negligible concentration, we did not quantify propene as part of the product distribution.

To ensure that the observed cracking and dehydrogenation products originated from the catalytic transformation of our substrate alkanes, we conducted additional control experiments. When carrying out our experiments on pure carbon paper, we did not observe any fragmentation or dehydrogenation of *n*-butane (Figures S10 and S11). Meanwhile, control experiments using pure metal loaded GDEs under argon (Ar) atmosphere showed minimal production of trace products (Figure S12), corresponding to less than 3% of the products observed when converting *n*-butane.

To rule out that the adsorption of impurities leads to the observed products, we separated the adsorption step from the dehydrogenation and fragmentation reaction. In a series of experiments, *n*-butane was first adsorbed onto the catalyst at 0.2 V vs SHE. Subsequently, the gas feed was switched to Ar, and after purging the cell for 10 min the potential was stepped to 0.5 V vs SHE and maintained for various hold durations (experimental details are provided in Figure S13). Through this experiment, we tested whether the observed products indeed originate from the electricity-driven fragmentation and dehydrogenation of preadsorbed *n*-butane. Our results (Figure S14a) show that the yields of C_1_, C_2_, and C_3_ products, as well as 1-butene, all increased over time, followed by a decrease at hold times over 15 min. This trend indicates that the products arise from surface reactions of adsorbed *n*-butane. At very long hold times, we saw a decrease in the amount of reductively desorbable products, which we attribute to the gradual oxidation of intermediates, as discussed previously.^13^ When carrying out the same experiments under Ar (Figure S14b), we saw only trace products amounting to less than 3% of the products observed in the presence of *n*-butane.

Our observed product distribution depended strongly on the potential applied during adsorption. Specifically, 1-butene and propane exhibited a common volcano-shaped trend between yield and adsorption potential, with a maximum at 0.5 V vs SHE. Ethane and ethylene also followed a common volcano-shaped trend, albeit with a peak at 0.3 V vs SHE. The fact that propane and 1-butene reached a maximum at more oxidative potentials than ethane and ethylene seems surprising at first, given that we have previously observed that alkane fragmentation becomes increasingly favorable at more oxidizing potentials.^12,13,15^ These trends provide insight into the possible interactions leading to fragmentation and dehydrogenation on the electrode surface.

We propose that the observed fragmentation product distribution may result from a potential-induced shift in *n*-butane fragment adsorption geometry (Figure S15). Specifically, *n*-butane undergoes dissociative adsorption (C–H dissociation and formation of a C–metal bond) to yield a C_4_H_8_ species bound to the surface via two carbon atoms. These adsorbed species can subsequently undergo C–C bond fragmentation between the two bound carbon atoms.^41,42^ Different adsorption configurations of the C_4_H_8_ species may therefore lead to differences in the fragmentation product distribution. Grand canonical density functional theory (GC-DFT) calculations^38–40^ support this hypothesis. At low potentials (<0.5 V vs SHE), our calculations led to similar adsorption energies for forming internally bound (β_1_,β_2_-C_4_H_8_) and terminally bound (α,β_1_-C_4_H_8_) *n*-butane fragments. However, terminal bonding becomes increasingly favorable over internal bonding at more positive potentials (Figure S16). In contrast to the regioselectivity of C–H bond fragmentation, the calculated C–C bond cleavage energies for *n*-butane fragments adsorbed to Pt(111) via terminal (α,β_1_-C_4_H_8_) and internal (β_1_,β_2_-C_4_H_8_) carbon atoms indicate that within the tested potential range, the cleavage energy is similar for both types of adsorbates (difference <0.05 eV). In addition, both energies remain nearly constant regardless of potential (Figure S17). We believe that these trends may explain why at lower potentials, products resulting from internal fragmentation are observed, while at higher potentials, we start to see more C_3_ products, resulting from terminal cleavage.

Methane likely results from consecutive fragmentation of intermediate fragments.^12,43^ Its yield reached a maximum at 0.5 V vs SHE. This is similar to our previous measurements using EC-MS, where we saw maximum methane generation at 0.6 V vs SHE at pH 0.^13^ Differences in product yields may be attributable to the increased local concentration of *n*-butane offered by GDEs, and to the different types of active sites offered by the sputtered Pt catalyst. When the applied potential exceeded 0.5 V vs SHE, the methane yield decreased, consistent with previous reports. This decrease can likely be ascribed to a reduction in the overall coverage of *n*-butane and an increased tendency for the overoxidation of reaction intermediates.^13,43,44^

We next sought to understand the impact of the catalyst material on the product distribution. Since forming strong metal–C bonds may promote C–C bond fragmentation, we reasoned that employing catalysts with weaker alkyl affinity than Pt might suppress adsorbate fragmentation and lead to higher dehydrogenation selectivity.^24,45,46^ To test this hypothesis, we repeated our experiments using palladium (Pd) instead of Pt as the catalyst material. The same electrolyte and potential sequence as for Pt catalysts were used. Our measurements (Figure 1c and Figures S18–21) indicated that *n*-butane conversion on Pd generated the same types of products as observed on Pt, pointing to similar mechanisms on either metal. The trends in adsorption and desorption current (Figures S22–S24) were in agreement with previous reports.^15,30^ Like on Pt, as the adsorption potential was increased on Pd, the yields of ethane/ethylene and propane/1-butene first rose and then declined, with propane and 1-butene peaking at more positive potentials. In contrast to Pt, however, Pd indeed displayed higher selectivity for 1-butene production compared to Pt (Figure S25), possibly because its weaker metal–C bond strength inhibits C–C bond cleavage yet preserves dehydrogenation activity.

In contrast to *n*-butane, the dehydrogenation of propane did not take place under our experimental conditions. We used GC-MS (Figure 3, Figures S26 and S27) to analyze the products obtained from adsorbing propane at two different potentials (0.3 and 0.5 V vs SHE) and releasing the adsorbates at 0.05 V vs SHE. Interestingly, despite the generation of fragmentation products, our GC-MS results revealed no detectable generation of propene. This observation may be attributed to the smaller enthalpy change required for *n*-butane dehydrogenation compared to propane,^4^ making the dehydrogenation reaction of *n*-butane thermodynamically more favorable at lower temperatures. Additionally, the stronger adsorption of *n*-butane on the Pt surface relative to propane may allow it to more readily undergo surface adsorption and subsequent conversion under identical experimental conditions.^46^ Evidence for this came from experiments with mixtures of *n*-butane and propane. Our data showed that the introduction of 5 vol % propane does not significantly alter the product yields observed under pure *n*-butane (Figure S28). This is likely attributable to the lower adsorption energy of propane compared to *n*-butane on Pt surfaces.^47^ When the propane concentration was increased to 20 vol %, a general decline in the yields of all products was observed (Figure S28), likely due to the dilution of the *n*-butane feed. These findings further suggest that adsorption and transformation of *n*-butane is preferred over propane, and that introducing propane does not lead to the generation of additional fragmentation products.

**Figure 3:**
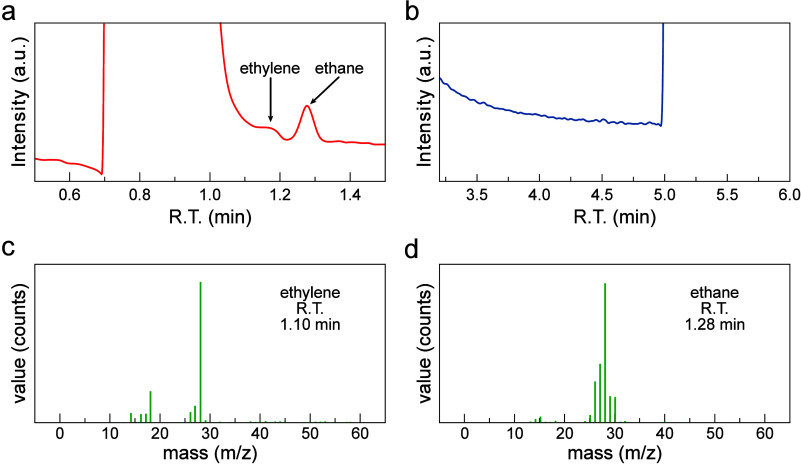
Results of GC-MS experiments of propane dehydrogenation on a Pt GDE. (a) TIC plots of ethylene and ethane peaks. (b) TIC plots of propene and propane peaks. GC-MS plots of (c) ethylene, (d) ethane corresponding to different retention times. The results show no detectable propene production. The adsorption potential was 0.3 V vs SHE. R.T., retention time. The complete TIC chromatogram can be found in Figure S26.

To improve the selectivity toward alkene production, we next modified the electrolyte composition. We considered that desorption may involve the displacement of surface adsorbates through the adsorption of *H to the catalyst.^24^ However, the presence of surface *H likely also leads to the hydrogenation of a fraction of the dehydrogenated adsorbates back to saturated alkanes.^48^ By increasing the electrolyte pH, we aimed to decrease the proton concentration at the electrode surface, and thereby suppress undesirable hydrogenation during the desorption step.^49,50^ To maintain the ionic strength, we replaced protons with Na^+^ ions and carried out our experiments in 0.1 M NaClO_4_ + 0.001 M HClO_4_. To compensate for the pH change, we maintained the adsorption and desorption potential constant vs the reversible hydrogen electrode (RHE) scale. We adsorbed *n*-butane at 0.3 V vs RHE, followed by desorption at 0.05 V vs RHE (Figure S29).

The results in Figure 4a show that replacing protons with Na^+^ cations enhanced the yield toward 1-butene production. This indicates that under the conditions of increased pH and increased alkali metal cation concentration, the dehydrogenated intermediates indeed have a higher propensity to desorb in the form of unsaturated products. Interestingly, however, we also observed an increase in propane production, but no longer detectable ethylene or ethane. This change in product distribution may be associated with a change to the bonding mode of *n*-butane fragments under the changed electrolyte conditions. Our calculations indicate (Figure S30) that in the presence of Na^+^, the terminally adsorbed *n*-butane fragment (α,β_1_-C_4_H_8_) exhibits increasingly favorable adsorption with increasing potential compared to the internally bound *n*-butane fragment (β_1_,β_2_-C_4_H_8_). Based on the discussion above, we propose that this preference for terminal bonding may lead to preferential fragmentation to C_1_ and C_3_ products (Figure 4b).

**Figure 4:**
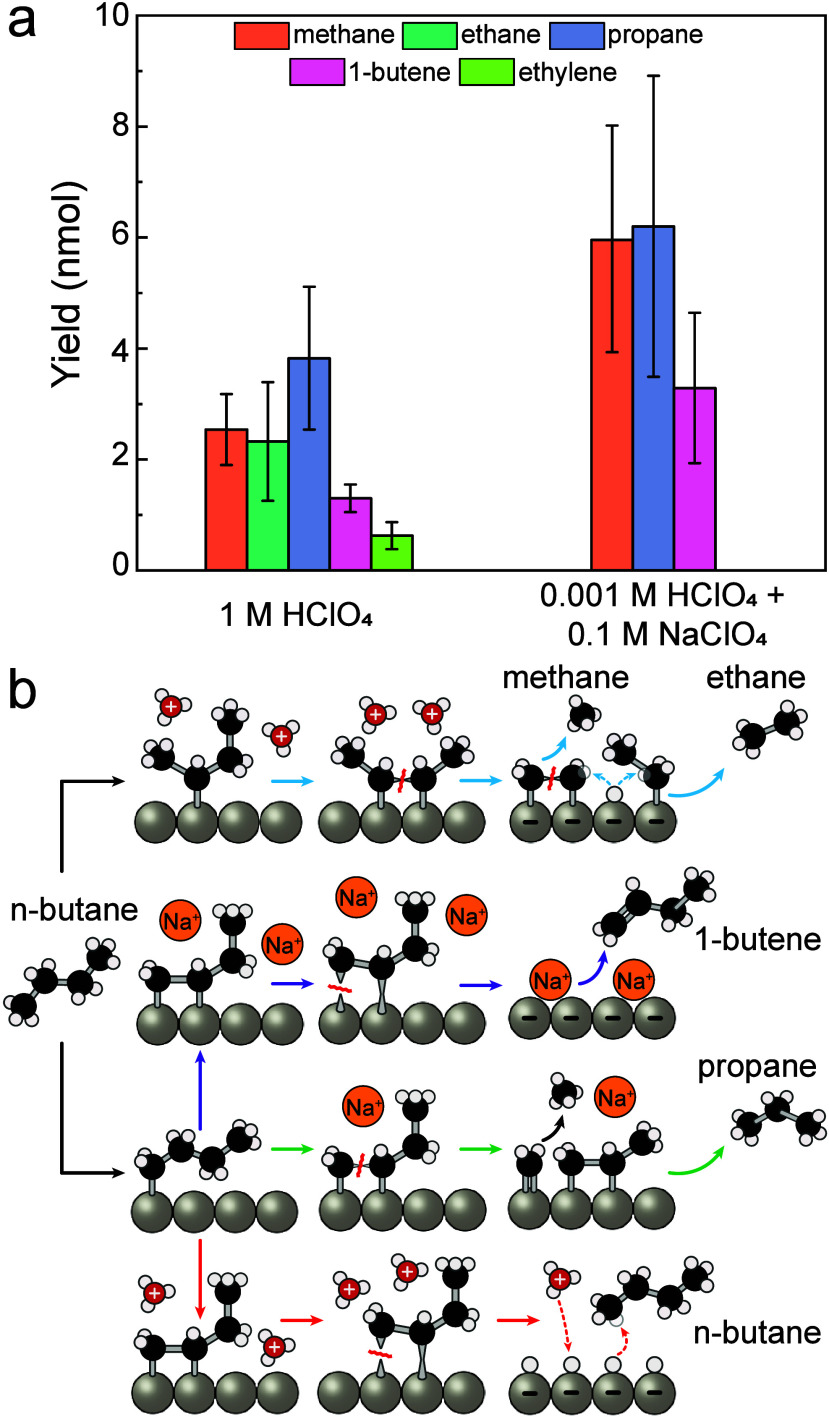
GC results of electrochemical *n*-butane dehydrogenation experiments in the presence of Na^**+**^ using Pt GDE. (a) GC results. Unconverted *n*-butane was not quantified. Three independent experiments were conducted using three GDE electrodes. Error bars represent standard deviations from triplicate measurements, with primary variability arising from slight differences in GDE catalyst loading. For experiments using 0.1 M NaClO_4_ and 0.001 M HClO_4_, the adsorption potential was 0.3 V vs RHE and the desorption potential was 0.05 V vs RHE. For experiment with 1 M HClO_4_, the adsorption potential was 0.3 V vs SHE and the desorption potential was 0.05 V vs SHE. (b) Proposed reaction pathways. For aqueous HClO_4_ conditions, the major surface reaction pathways are shown by the cyan and red arrows. For NaClO_4_-based electrolytes, the key pathways are indicated by the purple and green arrows. Atoms are not to scale.

Interestingly, the total yield of products increased when H^+^ ions were replaced with Na^+^. To verify if indeed more products were desorbed, we compared the total oxidation charge before (Q_adsorbed_) and after (Q_undesorbed_) the release of products using oxidative stripping experiments (Figure S31). This allowed us to quantify the fraction of undesorbed species. Our experimental results demonstrate a significant reduction in the ratio between the oxidation charge after and before product desorption upon exchanging H^+^ with Na^+^ ions, while also amplifying the difference in current intensity observed before and after reductive desorption compared to pure acid. (Figure S32). These results further support that the modified electrolyte was able to promote the formation of desorbable products and enhance product desorption.

In this work, we demonstrated a surface-catalyzed electrochemical route enabling the dehydrogenation of *n*-alkanes at room-temperature. Our findings lead the way to a sustainable alkane dehydrogenation process that can be driven by electrical energy. We hypothesize that the outcome of the reactions seen herein is enabled by the electrochemical dehydrogenative adsorption of alkane substrates, followed by the electricity-driven desorption of these dehydrogenated fragments. It is worth noting that while the proposed mechanism explains the product distribution, it will benefit from further investigation. For example, future research should clarify the molecular interactions through which alkali cations influence the bonding geometry of alkanes. Further investigation should also be directed at understanding how the electrolyte composition influences the degree of dehydrogenation of adsorbates and their molecular identity. In particular, adsorbed alkanes may undergo further dehydrogenation through repeated dissociations of C–H of bonds. Insight into these phenomena will require a combination of computational methods that can efficiently canvas complex reaction networks and in situ spectroscopic techniques that can identify dominant adsorbates on the surface. Moving the technology demonstrated herein toward industrial application will necessitate further enhancement of catalytic selectivity, which could be accomplished by further tuning the electrolyte alkalinity and metal cation concentration. In summary, our results highlight how time-dependent potential modulation enables challenging *n*-alkane transformations beyond C–C bond fragmentation and oxidation reactions, adding important building blocks on the way to designing the chemical industry of the future.

## Supplementary Material



## Notes

Additional figures, experimental details, and materials and methods, including photographs of the experimental setup, detailed descriptions of quantitative methods, quantitative results from control experiments, and raw gas chromatogram and GC-MS spectra. (PDF)
