# Supplementary material for: Room-Temperature Electrocatalytic Dehydrogenation and Partial Fragmentation of n‑Alkanes
Source: J Am Chem Soc. 2025 Sep 22;147(39):35219–26. doi: 10.1021/jacs.5c12806 (PMC12503361; doi:10.1021/jacs.5c12806)
Supplement: Supplementary file 1 [file ja5c12806_si_001.pdf]

Supporting Information

Room-Temperature Electrocatalytic Dehydrogenation and Partial Fragmentation of n-Alkanes

Gong Zhang,‡ Lee Fuller,† Christine Lucky,‡ Alexander J. Zielinski,† Dongfang Cheng,⊥ Enner Mendoza,‡ Philippe Sautet,⊥§# Marcel Schreier‡†*

‡ Department of Chemical and Biological Engineering, University of Wisconsin–Madison, Madison, WI 53706, USA

† Department of Chemistry, University of Wisconsin–Madison, Madison, WI 53706, USA.

⊥ Department of Chemical and Biomolecular Engineering, University of California, Los Angeles, CA 90095, USA

§ Department of Chemistry and Biochemistry, University of California, Los Angeles, CA 90095, USA

# California NanoSystems Institute, Los Angeles, CA 90095, USA.

Table of Contents

1. Experimental Procedures for Gas Chromatography (GC)

1.1 Materials

1.2 Electrochemical Measurements

1.3 GC Calibration

1.4 Collection of Desorbed Products

2. Experimental Procedures for Gas Chromatography-Mass Spectrometry (GC-MS)

2.1 Materials

2.2 Electrochemical Measurements

3. Theoretical Calculations

4. Supplemental Figures

5. References

1. Experimental Procedures for Gas Chromatography (GC)

1.1 Materials

Carbon paper (H15C13, Freudenberg) was used as the electrode substrate. The catalyst material was deposited on the substrates using a sputter coater (Anatech USA), with the sputtering target (platinum and palladium, 99.9%) purchased from ACI Alloys, Inc. The electrolyzer used was a custom-built cell, as shown in **Figure S2 and S3**. The liquid compartment was constructed from polymethyl methacrylate (PMMA), and was cleaned in a laboratory dishwasher (PLW 8505, Miele Professional). The gas compartment is made of Ti (Grade 2, McMaster-Carr) and features a serpentine flow channel. The back side of the GDE (non-catalyst side) is in direct contact with the metal to ensure good electrical conductivity. A polytetrafluoroethylene (PTFE) gasket was used to achieve a gas-tight seal for the system. All water used for electrolyte preparation was from a Milli-Q® Integral Water Purification System (EMD Millipore). Electrolytes were prepared with perchloric acid ($HClO_4$, 70%, 99.999% trace metals basis, Sigma-Aldrich). Argon gas (Ar, UHP grade, Airgas) was used as a purge gas for all electrolyte solutions and was also employed as carrier gas for GC measurements during the experiments and calibration. Propane and n-butane gases (Research grade, 99.99%) were purchased from Airgas.

1.2 Electrochemical Measurements

A Gamry Interface 1010E potentiostat was used for all electrochemical measurements. Potentiostatic electrochemical impedance spectroscopy (PEIS) was used to determine the uncompensated resistance (R_u) of the electrochemical cell by applying voltage waveforms about the open circuit potential with an amplitude of 10 mV and frequencies ranging from 10 Hz to 100 kHz. The potentiostat compensated for 85% of R_u in-situ to arrive at accurate potentials. A leakless miniature Ag/AgCl (3.4 M KCl, eDAQ Inc) was employed as reference electrode. Unless otherwise noted, 1 M $HClO_4$ was used as electrolyte. The electrolyte was sparged with Ar (100 standard cubic centimeters per minute, sccm) for at least 30 minutes prior to experiments to remove oxygen before entering the electrochemical cell. Before all experiments, the working electrode (Pt or Pd GDE) was conditioned by applying 1.3 V *vs* the standard hydrogen electrode (SHE, all potentials are relative to SHE unless otherwise noted) for 5 min followed by 10 cyclic voltammetry (CV) cycles between −0.1 and 1.6 V at a scan rate of 50 mV s^{-1}. Before applying the adsorption potential, the system was stabilized at the open-circuit potential for 5 min, during which n-butane or propane was introduced into the electrolyzer at a flow rate of 10 sccm. The alkane adsorption step always consisted of a 15 min hold at a given potential under pure n-butane or pure propane flow (10 sccm) followed by 15 min at the same potential under Ar flow to remove residual substrate from the system. During the first 10 min of the Ar flow, the gas flow rate was 60 sccm, then decreased to 1 sccm before desorption. Simultaneously, fresh electrolyte was flowed at 1 mL/min using a syringe pump (Model 22, Harvard Apparatus), and the flow was stopped 1 min prior to desorption. The gas flow rate was controlled using a mass flow controller (MFC, Alicat, MC series). For desorption, the potential was immediately stepped to 0.05 V to remove cathodically desorbable surface species. The procedure for collecting and analyzing products is described below.

For experiments with propane and n-butane mixtures, we used 1 M $HClO_4$ (pH 0) as the electrolyte. Adsorption was performed at potentials of 0.3 V and 0.5 V *vs* SHE, followed by a desorption step at 0.05 V *vs* SHE. Different propane/n-butane mixture concentrations were achieved by regulating the respective gas flow rates using two mass-flow controllers (Alicat, MC series). The outlets of the two MFCs were connected *via* a tee, and the mixed gas was then introduced into the gas compartment of the electrolytic cell. For example, a 5 vol% propane in n-butane mixture was obtained by maintaining a total flow rate of 50 sccm, comprising 2.5 sccm of propane (Research Grade, 99.99%, Airgas) and 47.5 sccm of n-butane (Research Grade,

99.99%, Airgas). All other experimental conditions were kept consistent with those of all other experiments.

Potentiostatic electrochemical impedance spectroscopy (PEIS) was used to determine the uncompensated resistance (R_u) of the electrochemical cell by applying voltage waveforms about the open circuit potential with an amplitude of 10 mV and frequencies ranging from 10 Hz to 100 kHz. The potentiostat compensated for 85% of R_u in-situ to arrive at accurate potentials.

1.3 GC Calibration

Gas products (methane, ethylene, ethane, propene, propane, 1-butene, n-butane) were quantified using an SRI gas chromatography system (Multiple Gas Analyzer #5, SRI Instruments). The flame ionization detector (FID) was connected to a Hayesep D packed column (12' ×1/8", SRI Instruments) to detect hydrocarbons. Ar was used as carrier gas.

Product quantification was accomplished by using calibration curves. Calibration curves were obtained by injecting various volumes of a standard gas mixture (Airgas, standard gas mixture balanced by Ar) into the GC. A gas-tight syringe was used to collect various volumes of the standard gas mixture (3 mL, 2 mL, 1 mL, 0.5 mL). This aliquot of the gas mixture was then injected onto the Hayesep D column. The standard gas mixture included n-butane (998.2 ppm), 1-butene (49.75 ppm), propane (1011 ppm), propene (50.30 ppm), ethane (1011 ppm), ethylene (50.67 ppm), and methane (1011 ppm). By knowing the concentrations of the gaseous components in the standard gas mixture and the volume of the gas injection into the GC, peak areas of the gaseous components were converted to nmol quantities by assuming the ideal gas law.

1.4 Collection of Desorbed Products

Before starting the product desorption, we connected a gas-tight syringe (10 mL, Micro-Mate Interchangeable Syringes, Cadence Science) with a Luer fitting to the Luer port at the outlet of the cell's gas compartment (**Figure S3**). During product collection, the MFC was set to flow control mode with a target Ar flow rate of 1 sccm. Once the potential was switched to the desorption potential, the valve connected to the syringe was opened, allowing Ar to slowly carry the products into the syringe, which automatically drives the plunger forward. This process typically required a volume of 6 mL to collect all desorbed products. To prevent contamination, the GDE was heated in air to 350°C after each experiment to remove any residual adsorbates on the carbon paper.[1]

2. Experimental Procedures for Gas Chromatography-Mass Spectrometry (GC-MS)

2.1 Materials

The materials used were identical to those used in the GC tests described above, except that helium (He, UHP grade, Airgas) was used as the carrier gas in the GC-MS analysis.

2.2 Electrochemical Measurements

The electrochemical test method was identical to that used in the GC experiments (**Section 1.2**).

2.3 GC-MS Setup

The sample collection method was identical to that used in the GC experiments (**Section 1.4**). Product analysis was carried out by GC-MS (ISQ™ 7610 Single Quadrupole GC-MS, Thermo Fisher Scientific Inc.) equipped with a capillary column (ValcoPLOT® Capillary Column Hayesep-D, 15 m × 0.32 mm × 10 μm, VICI Metronics). A split/splitless injector was used, with the split ratio maintained at 2. The column was held at 60°C for 45 min, and the flow of the carrier (helium) was 1 ml min^{-1}. The temperature of the injector, ion source, and MS Transfer line was set to be 110, 300, and 300°C, respectively. The mass-to-charge ratio of the mass scanning mode was set from 1 to 60. The background signal of the GC-MS instrument is shown in **Figure S9**.

3. Theoretical Calculations

Density functional theory (DFT) calculations were performed using the plane-wave based Vienna Ab Initio Simulation package (VASP).[2] The electronic exchange and correlation effects were described by the generalized gradient approximation (GGA) in the form of the revised Perdew-Burke-Ernzerhof (RPBE) functional.[3] The D3 correction method was employed to illustrate the long-range dispersion interactions between the water and surface.[4] The interaction between atomic cores and electrons was described by the projector augmented wave (PAW) method.[5] A cutoff energy of 400 eV for the plane-wave basis set and an atomic force convergence for geometry optimization of 0.02 eV/Å were employed. The Pt(111) surface was modeled by a 3-layer (4 × 4) supercell. The bottom layer of the slab was constrained as the bulk region, and the top two layers and adsorbates were allowed to relax as the interface region. The Brillouin zone was sampled using the 4 × 4 × 1 Gamma-centered k-point grids. The Gibbs free energies for adsorbates on the surface is obtained from the DFT energies with zero-point energy (ZPE) and entropy corrections determined from frequency calculations performed for all the considered structures using the harmonic oscillator approximation. The linearized Poisson Boltzmann implicit solvation model implemented in VASPsol is used to represent the implicit electrolyte region.[6] The dielectric constant of water, 78.4, and the Debye screening length of 3.0 Å, were used. For calculations in the presence of Na^+, an explicit Na^+ atom was included in the optimization. While explicitly considering the presence of the cation, we also employed an implicit solvation model to describe the solvation of the cation near the surface. The reliability of this optimization method has been described in detail in our previous research.[7, 8]

Grand Canonical DFT (GC-DFT) calculations are performed to obtain the potential-dependent energetics, where the number of electrons is allowed to change to adapt to the change of work function along the reaction pathway. The potential-dependent grand canonical free energy is expressed within the surface charging model as follows:

$$\Omega(U) = \Omega(U) - q(U) \cdot F(U) = \Omega(U_0) - \frac{1}{2}C(U - U_0)^2$$

where $\Omega(U)$ is the electronic energy of the surface at the U potential in the SHE scale, $q(U)$ is the charge difference against the neutral condition, F is the Faradaic constant. C stands for the effective capacitance and U_0 represents the potential of zero charge (pzc). C and U_0 are obtained by fitting the quadratic relationship of the energies as the function of potential. For more details of GCDFT, see our previous work.[7, 9-11]

Given that our gas diffusion electrodes (GDEs) were fabricated using conventional sputtering without capping agents, the predominant exposed surface is expected to correspond to the thermodynamically most stable facet, namely Pt(111). This expectation aligns with literature calculations indicating that Pt(111) possesses the lowest surface energy within our experimental potential window (0.2–0.7 V *vs* SHE).[12] Based on this reasoning, we selected this surface for our computational modeling.

However, we certainly acknowledge that in a real electrode system, n-butane adsorption may also occur at other types of sites, such as defects, step edges, or different low-index facets that might be present. Therefore, it is important to emphasize that our calculations, which focus on the idealized Pt(111) surface, are intended primarily to provide qualitative insights and a potential mechanistic rationale for the observed experimental trends. They should not be interpreted as aiming for a precise quantitative prediction of the overall behavior of the complex polycrystalline electrode.

4. Supplemental Figures

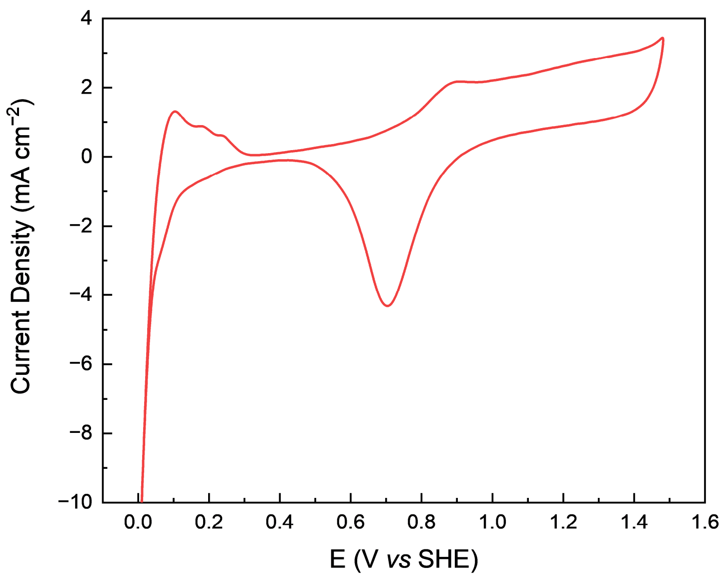

Figure S1. A representative cyclic voltammogram of the Pt GDE. This test was done using our custom-built GDE cell (**Figure S2**). During the measurement the electrolyte was stagnant 1 M $HClO_4$ and Ar was supplied at a flow rate of 60 sccm. The scan rate was 10 mV/s. The CV is in agreement with previous reports.[13, 14]

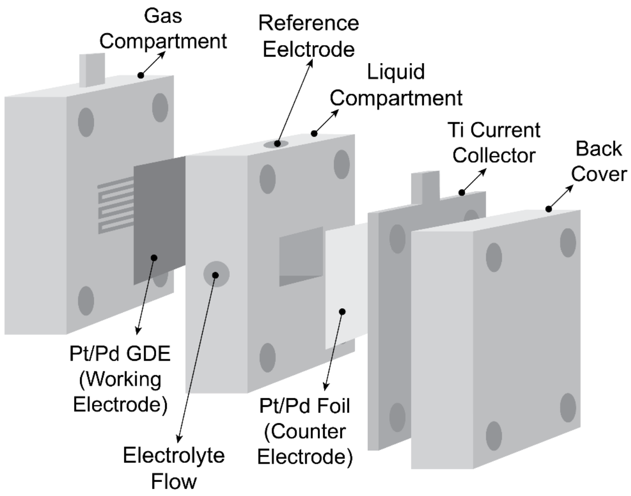

Figure S2. Illustration of the electrochemical cell. The electrolyzer consists of two compartments: a gas compartment and a liquid compartment. The liquid compartment does not contain any internal flow channel structures. The detailed cell assembly procedure has been described in previous reports.[15] The working and counter electrode contained in the same electrolyte, and no membrane was employed. The exposed geometric area of both the working and counter electrodes was 1 cm^2.

a b
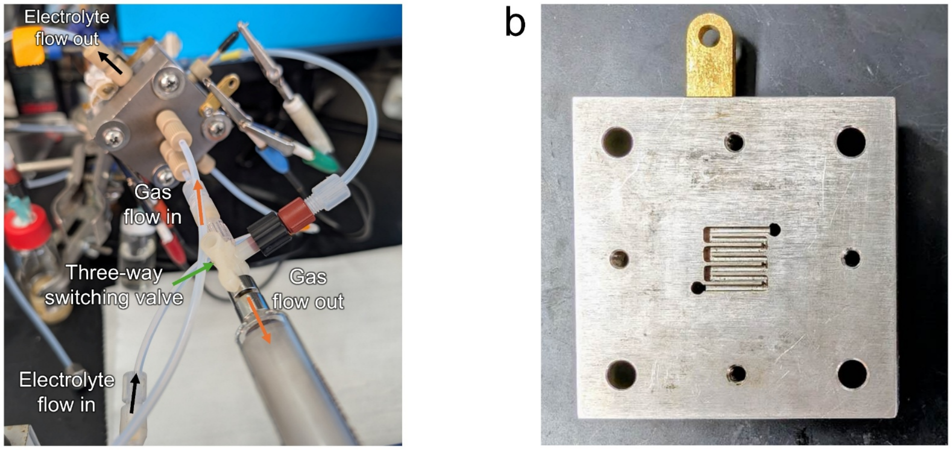

Figure S3. Experiment setup. (a) Photograph of the assembled electrochemical cell. **(b)** Photograph of the gas compartment with a serpentine flow channel.

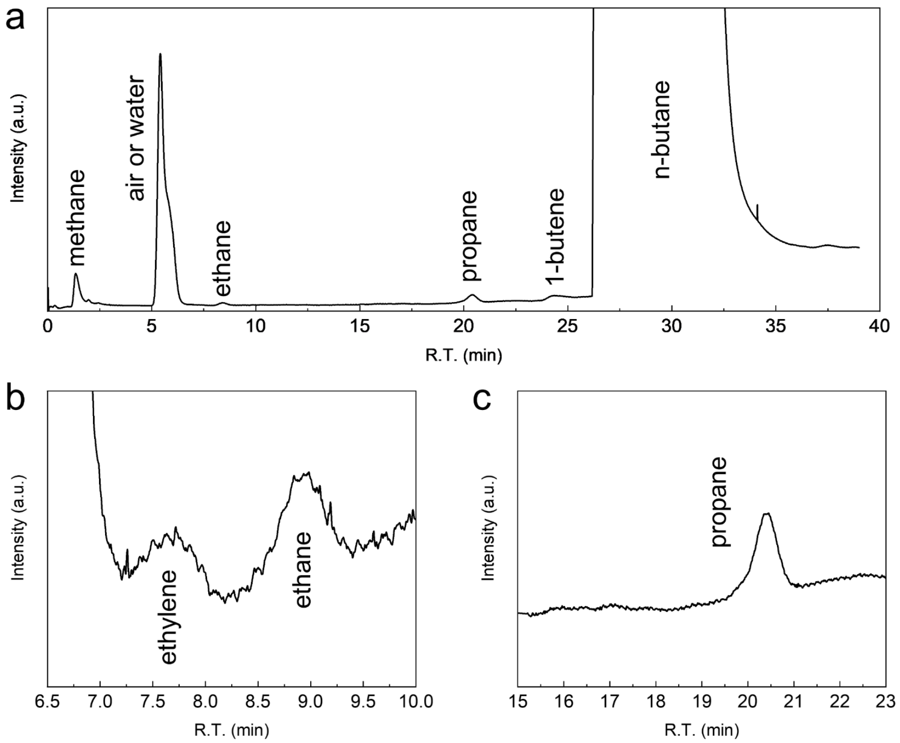

Figure S4. A typical gas chromatogram resulting from n-butane conversion on a Pt GDE with an adsorption potential of 0.3 V *vs* SHE. (a) The full-range result. **(b)** Detail corresponding to the ethylene and ethane peaks. **(c)** Detail corresponding to the propane peak.

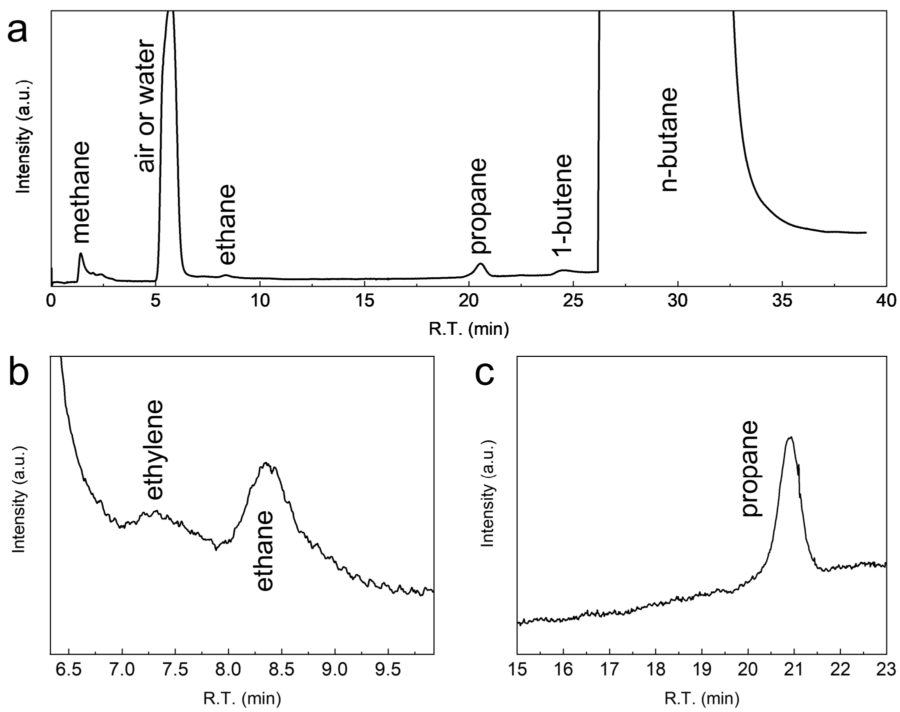

Figure S5. A typical gas chromatogram resulting from n-butane conversion on a Pt GDE using an adsorption potential of 0.5 V *vs* SHE. (a) The full-range result. **(b)** Detail corresponding to the ethylene and ethane peaks. **(c)** Detail corresponding to the propane peak.

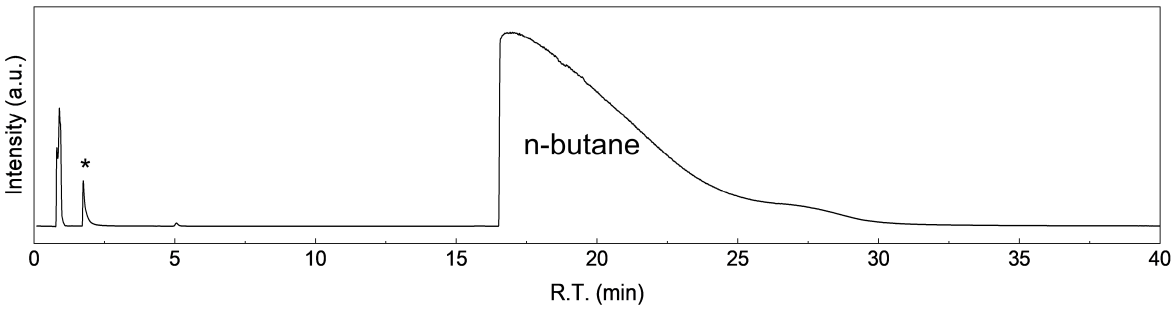

Figure S6. Total ion chromatography (TIC). The adsorption potential for this measurement was 0.5 V *vs* SHE. This figure is related to **Figure 2** in the main text. The peak with * may be the signal of water, and the corresponding MS result is shown in **Figure S8**.

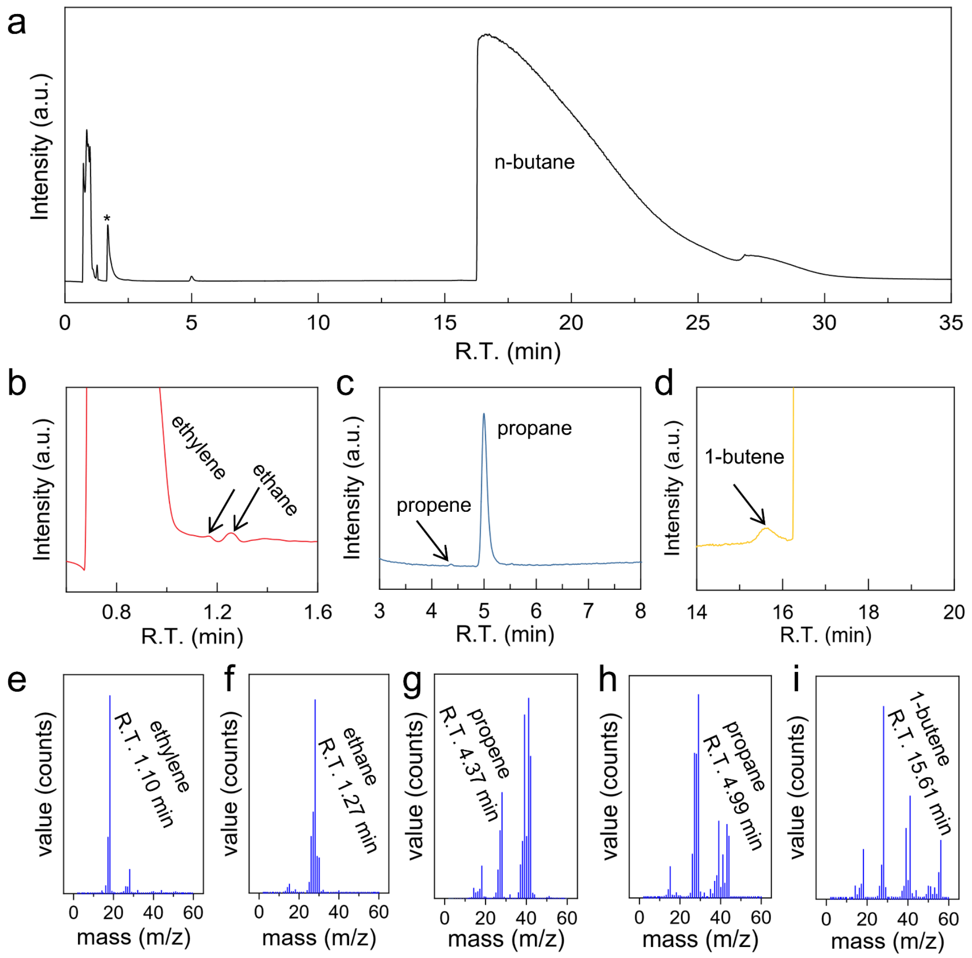

Figure S7. Results of GC-MS experiments of n-butane conversion on a Pt GDE. (a) Total ion chromatography (TIC). **(b)** Zoom-in plots of ethylene and ethane peaks. **(c)** Zoom-in plots of propene and propane peaks. **(d)** Zoom-in plots of the 1-butene peak. MS plots of **(e)** ethylene, **(f)** ethane, **(g)** propene, **(h)** propane, **(i)** 1-butene corresponding to different retention times. The adsorption potential was 0.3 V *vs* SHE. R.T. indicates retention time. The peak with * may be the signal of water, and the corresponding MS result is shown in **Figure S8**. The background signal of the instrument can be found in **Figure S9**.

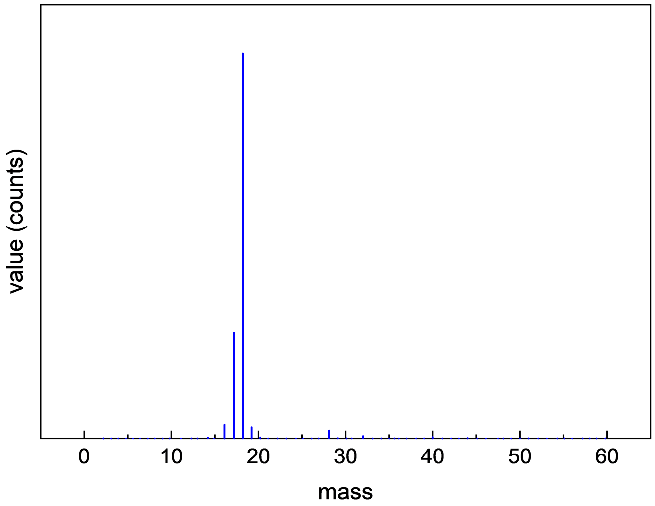

Figure S8. The MS signal corresponding to the suspected water signal observed in GC-MS.

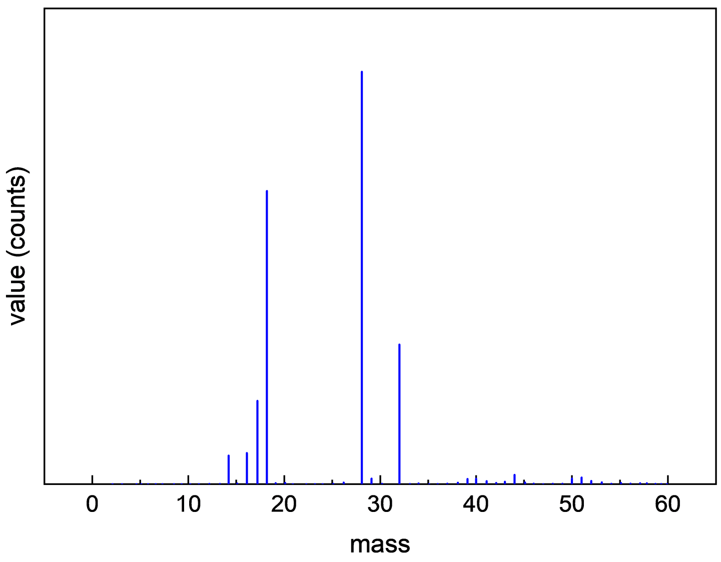

Figure S9. Instrument background signal of GC-MS.

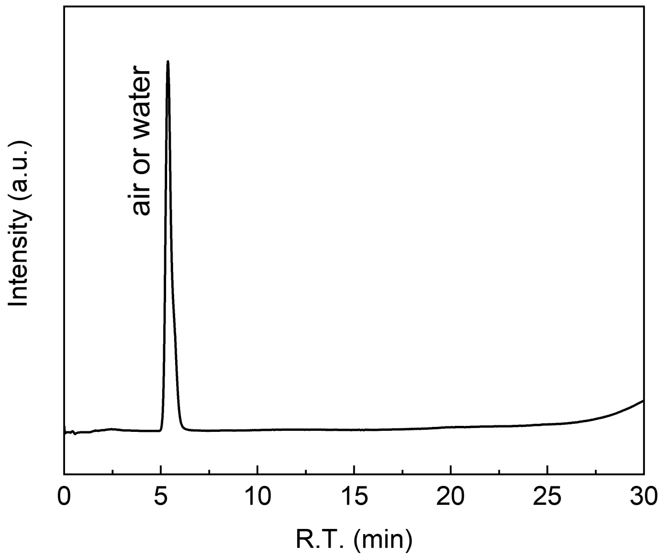

Figure S10. Typical gas chromatogram of n-butane conversion using blank carbon paper. The adsorption potential was 0.5 V *vs* SHE. The result showed no significant cracking or dehydrogenation products, indicating that the carbon paper itself does not exhibit catalytic properties.

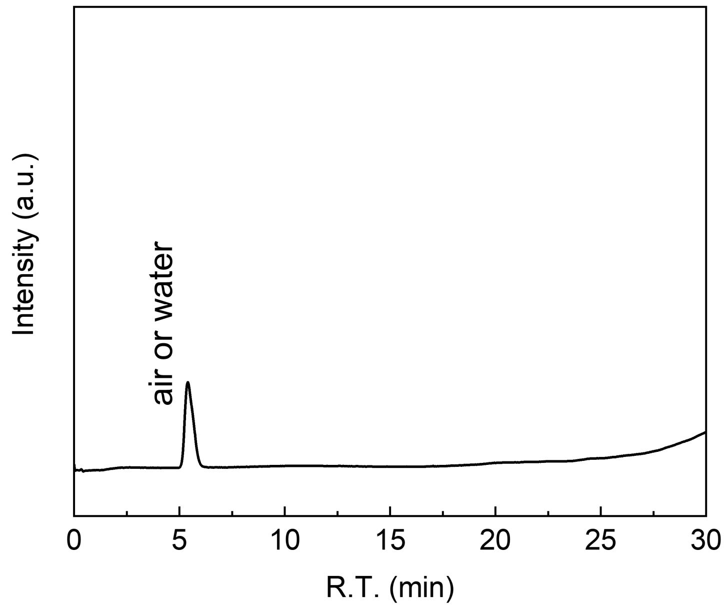

Figure S11. Control experiment using blank carbon paper under Ar atmosphere. The adsorption potential was 0.5 V *vs* SHE. The result showed that there was almost no detectable carbonaceous products formation.

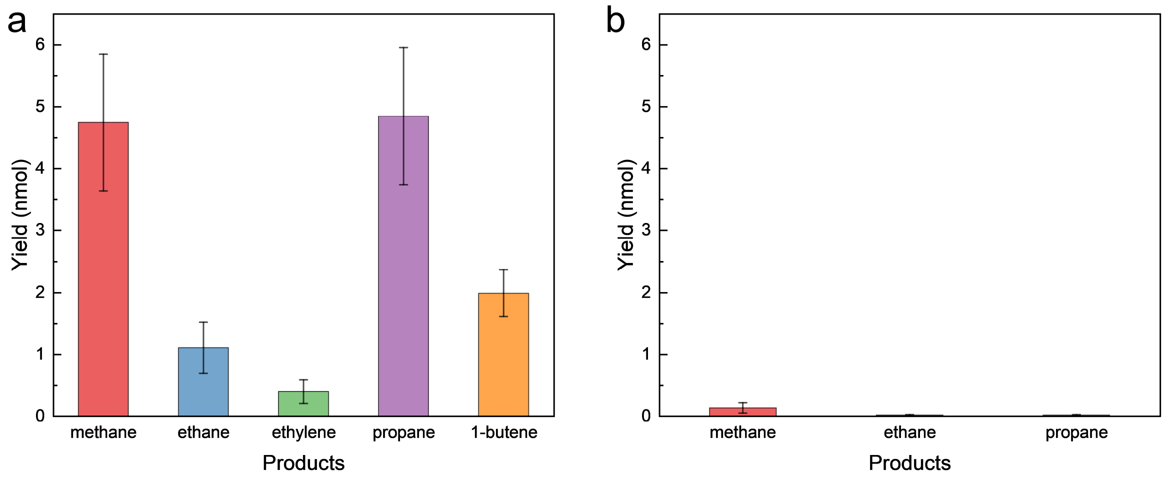

Figure S12. Control experiment using Pt GDE. (a) Experiments under n-butane. **(b)** Experiments under Ar. The adsorption potential was 0.5 V *vs* SHE. Notably, no alkene products were detected under Ar atmosphere. Three independent experiments were conducted using three GDEs to obtain error bars. Error bars represent standard deviations from triplicate measurements, with primary variability arising from slight differences in GDE catalyst loading.

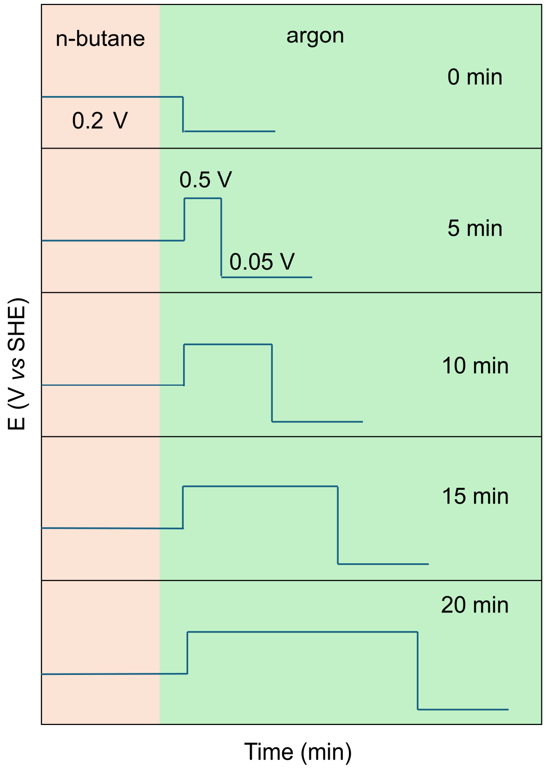

Figure S13. Potential profile for the transformation of pre-adsorbed n-butane. Under an n-butane atmosphere, the potential was held at 0.2 V *vs* SHE for 15 minutes. After switching the atmosphere to Ar, the potential was held at 0.2 V *vs* SHE for 5 minutes, then 0.5 V *vs* SHE was applied for varying durations. The desorption potential was 0.05 V *vs* SHE. The working electrode was a Pt GDE.

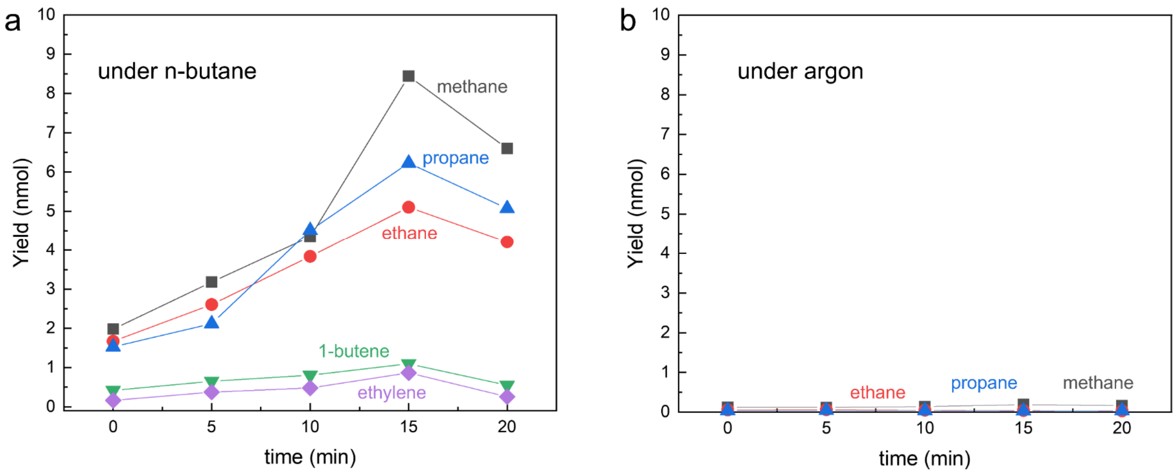

Figure S14. Product yields upon reductive desorption after holding at 0.5 V *vs* SHE of varying durations. (a) Experiments under n-butane. **(b)** Experiments under Ar. Notably, no alkene products were detected under the atmosphere. Points represent the average of two trials. The working electrode was a Pt GDE.

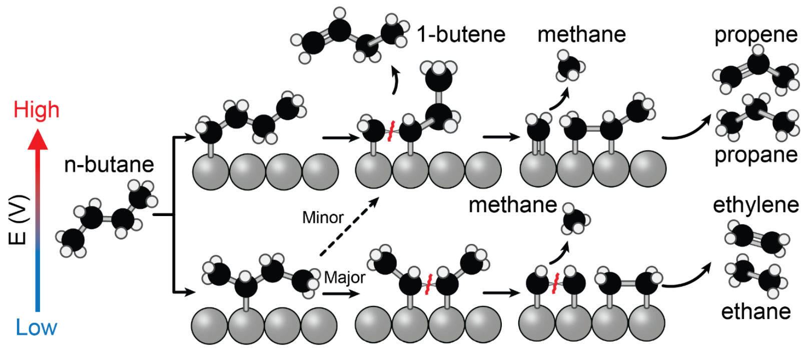

Figure S15. Hypothetical reaction mechanism for alkane dehydrogenation and fragmentation.

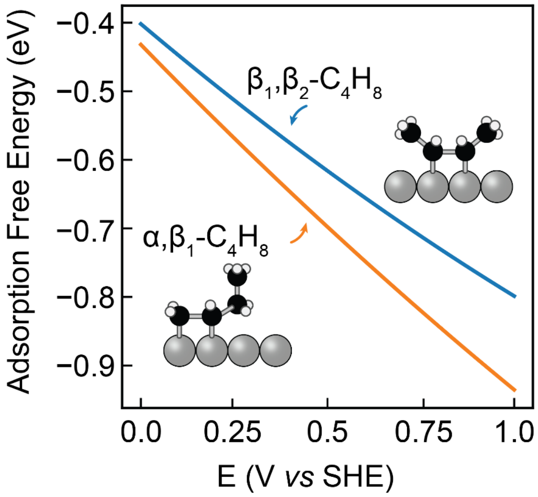

Figure S16. DFT-derived adsorption energy of n-butane fragment species (C_4H_8) as a function of potential.

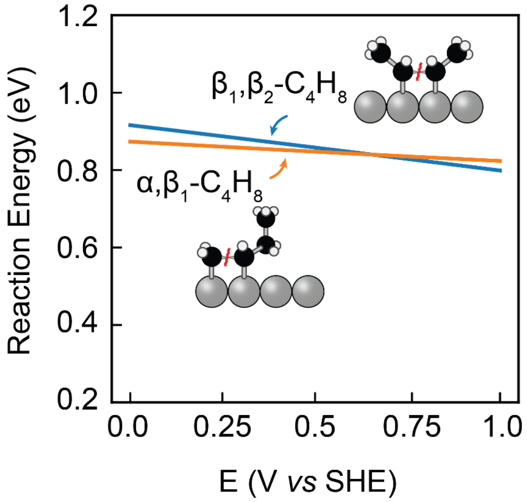

Figure S17. Effect of potential on the C–C bond cleavage energy of n-butane fragment species (C_4H_8) of different bonding configuration.

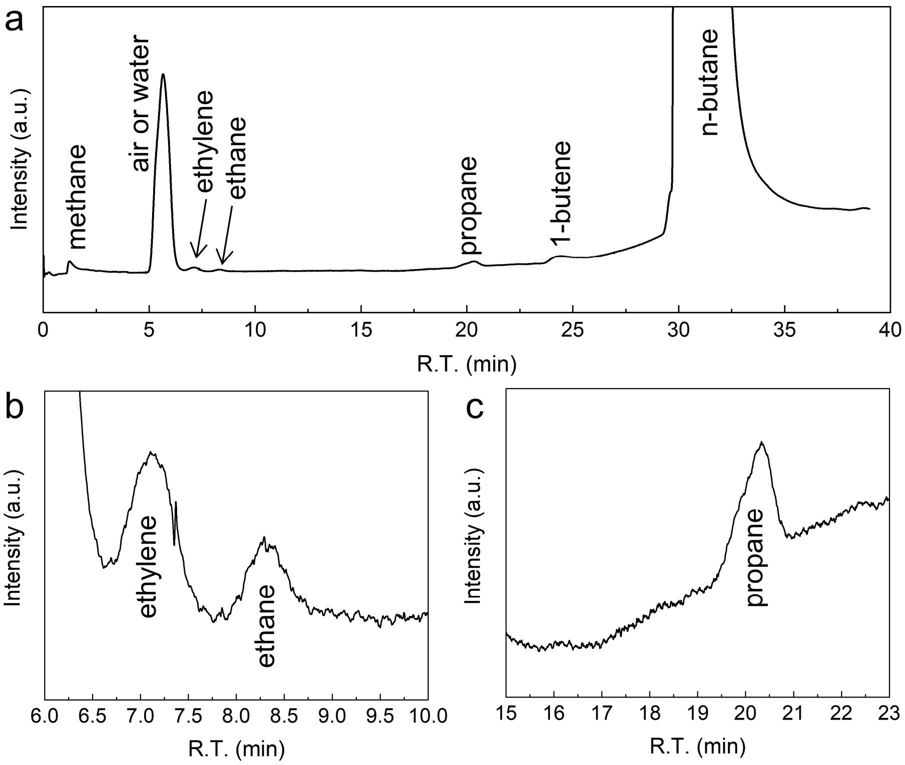

Figure S18. A typical gas chromatogram of n-butane conversion on a Pd GDE using an adsorption potential of 0.3 V *vs* SHE. (a) The full-range result. **(b)** Detail corresponding to the ethylene and ethane peaks. **(c)** Detail corresponding to the propane peak.

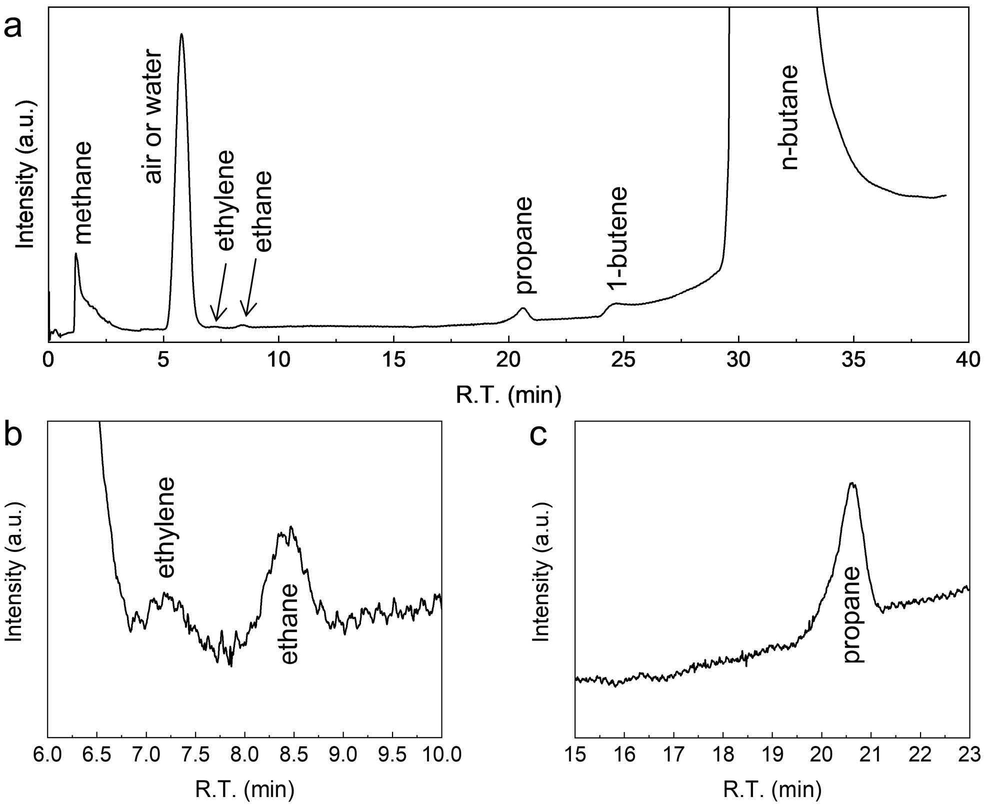

Figure S19. A typical gas chromatogram of n-butane conversion on a Pd GDE using an adsorption potential of 0.5 V *vs* SHE. (a) The full-range result. **(b)** Detail corresponding to the ethylene and ethane peaks. **(c)** Detail corresponding to the propane peak.

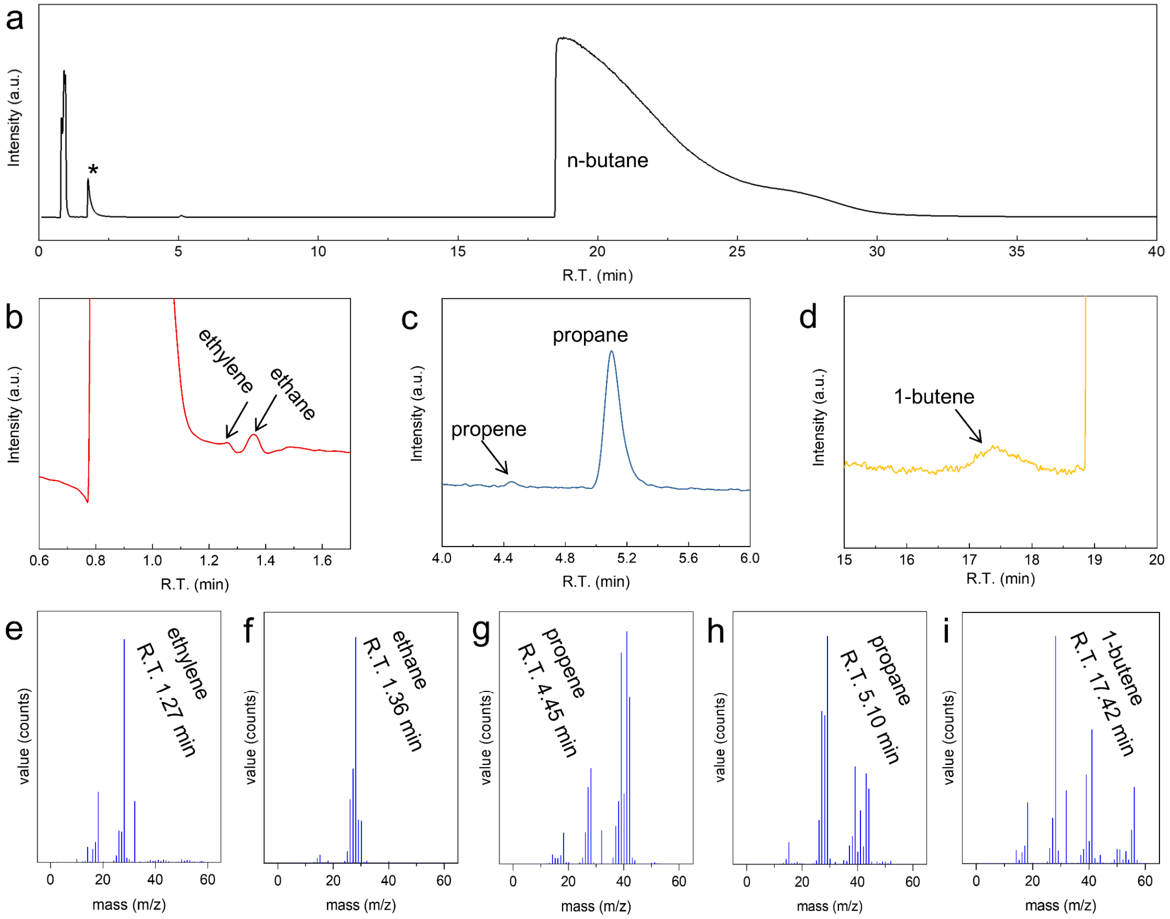

Figure S20. Results of GC-MS experiments of n-butane conversion on a Pd GDE. (a) Total ion chromatography (TIC). **(b)** Zoom-in plots of ethylene and ethane peaks. **(c)** Zoom-in plots of propene and propane peaks. **(d)** Zoom-in plots of the 1-butene peak. MS plots of **(e)** ethylene, **(f)** ethane, **(g)** propene, **(h)** propane, **(i)** 1-butene corresponding to different retention times. The adsorption potential was 0.3 V *vs* SHE. R.T. indicates retention time. The peak with * may be the signal of water, and the corresponding MS result is shown in **Figure S8**. The background signal of the instrument can be found in **Figure S9**.

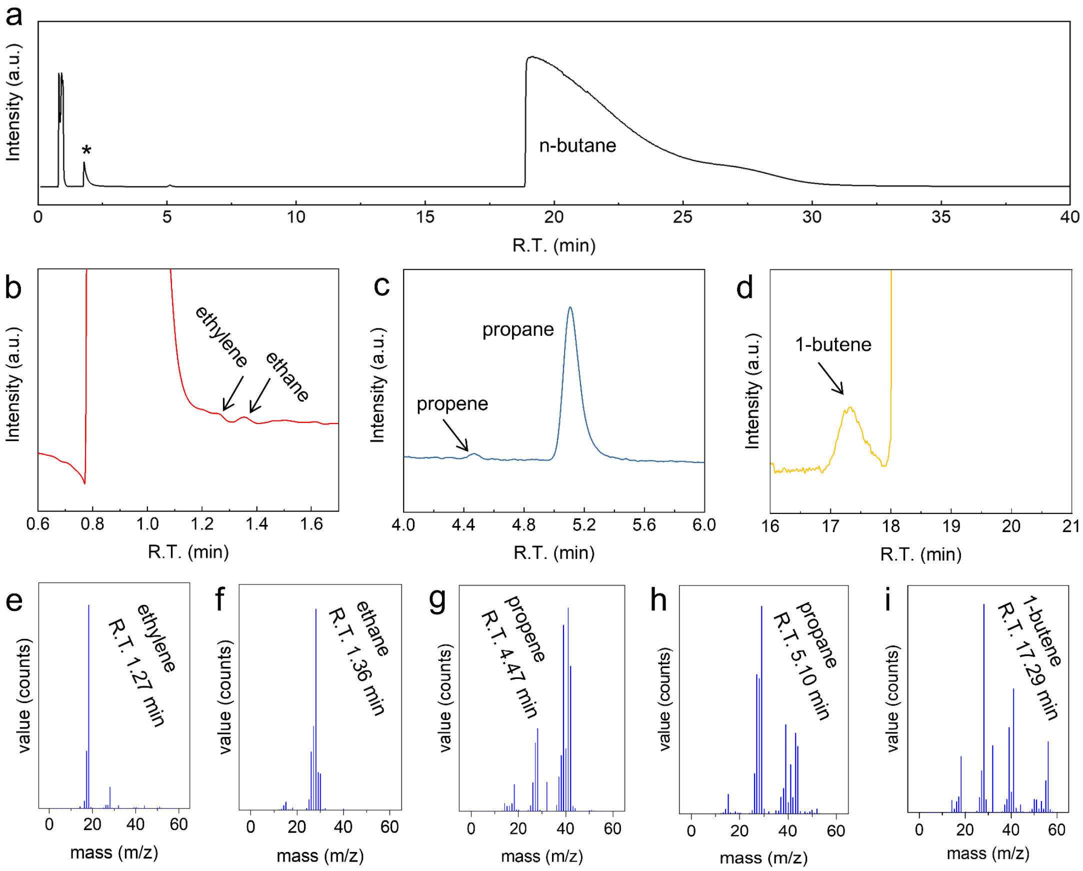

Figure S21. Results of GC-MS experiments of n-butane conversion on a Pd GDE. (a) Total ion chromatography (TIC) of GC-MS. **(b)** Zoom-in plots of ethylene and ethane peaks. **(c)** Zoom-in plots of propene and propane peaks. **(d)** Zoom-in plots of the 1-butene peak. MS plots of **(e)** ethylene, **(f)** ethane, **(g)** propene, **(h)** propane, **(i)** 1-butene corresponding to different retention times. The adsorption potential is 0.5 V *vs* SHE. R.T. indicates retention time. The peak with * may be the signal of water, and the corresponding MS result is shown in **Figure S8**. The background signal of the instrument can be found in **Figure S9**.

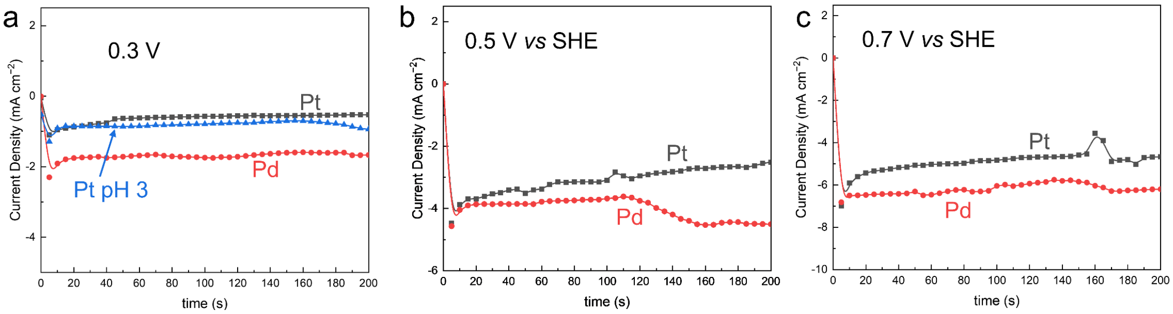

Figure S22. Current measured during desorption. (a) Desorption current after adsorption at 0.3 V *vs* the reversible hydrogen electrode (RHE, =0.3 V *vs* SHE at pH 0). **(b)** Desorption current after adsorption potential at 0.5 V *vs* SHE. **(c)** Desorption current after adsorption at 0.7 V *vs* SHE.

We observed slight variations in the desorption current under different GDEs and adsorption potential conditions. These differences may result from variations in hydrogen evolution reaction (HER) performance or hydrogen intercalation activity.[13, 16]

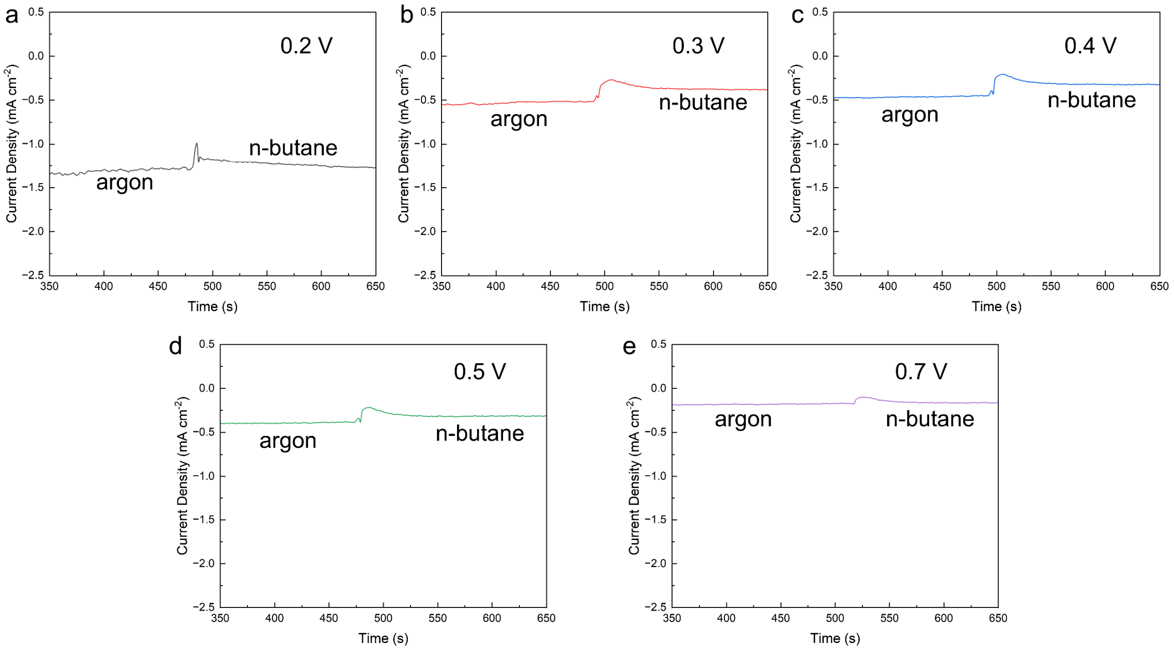

Figure S23 Current measured during adsorption on the Pt GDE. The electrolyte used at all adsorption potentials was 1 M $HClO_4$. All potentials are referenced to SHE, unless otherwise specified.

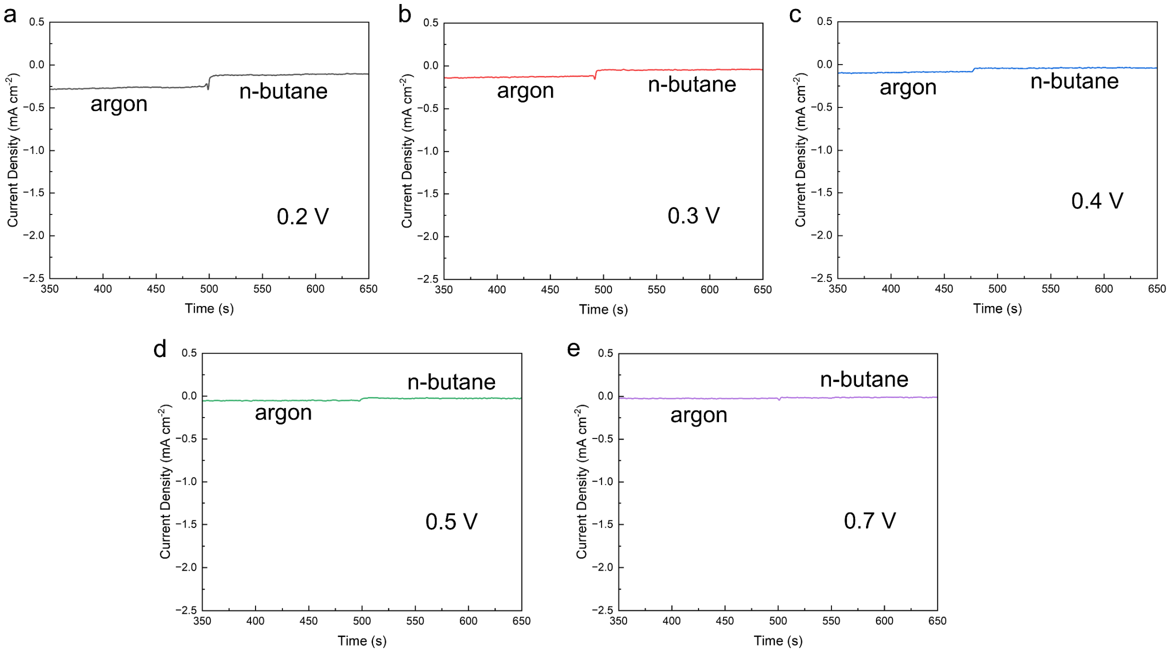

Figure S24 Current measured during adsorption on the Pd GDE. The electrolyte used at all adsorption potentials was 1 M $HClO_4$. All potentials are referenced to SHE, unless otherwise specified.

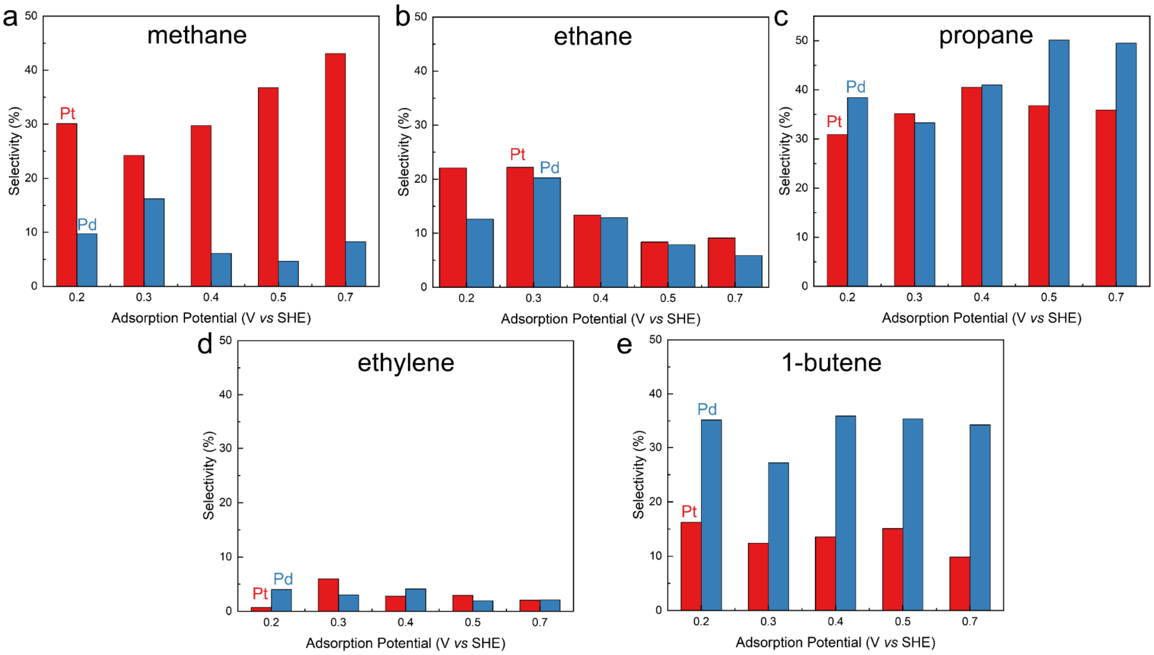

Figure S25. Product selectivity of n-butane conversion on Pt and Pd GDEs at various adsorption potentials. This figure is related to **Figure 1** in the main text.

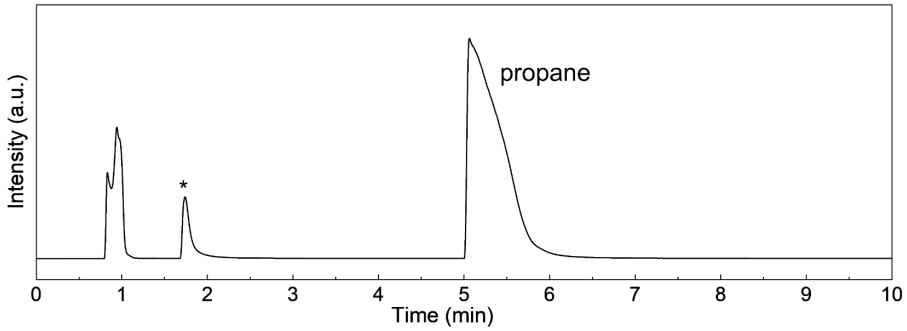

Figure S26. Total ion chromatography (TIC). The adsorption potential for this measurement was 0.3 V *vs* SHE. This figure is related to **Figure 3** in the main text.

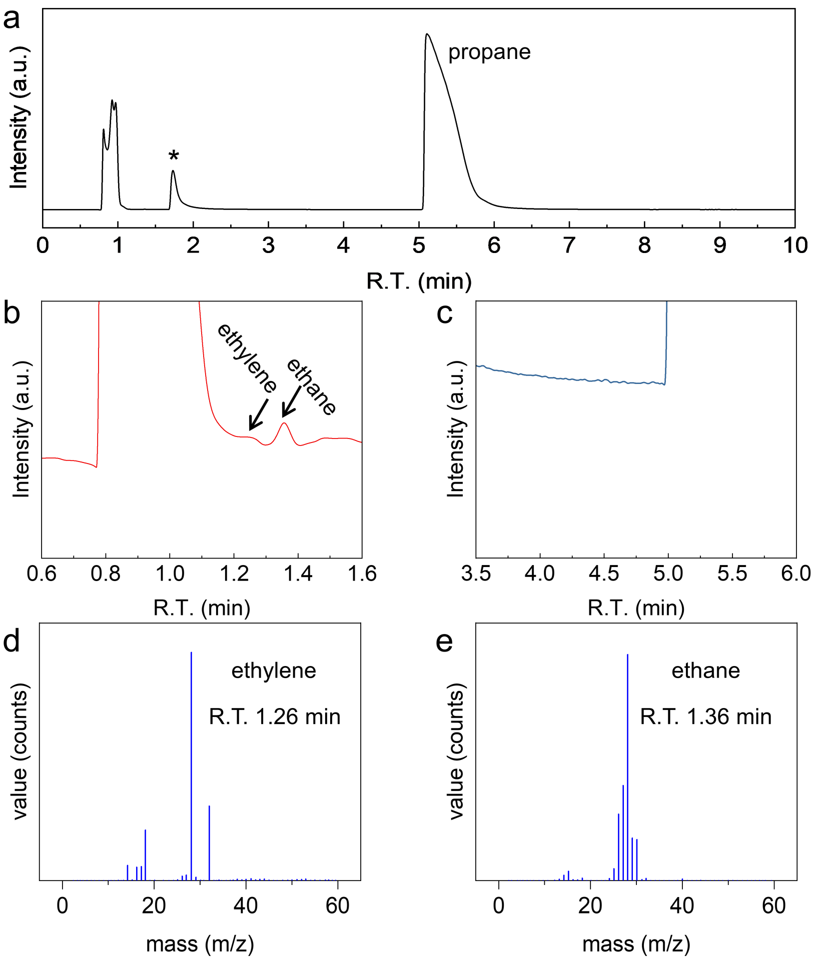

Figure S27. Results of GC-MS experiments of propane conversion on a Pt GDE. (a) Total ion chromatography (TIC). **(b)** Zoom-in plots of ethylene and ethane peaks. **(c)** Zoom-in plots of propene and propane peaks. MS plots of **(d)** ethylene, **(e)** ethane. The adsorption potential is 0.5 V *vs* SHE. R.T. indicates retention time. The peak with * may be the signal of water, and the corresponding MS result is shown in **Figure S8**. The background signal of the instrument can be found in **Figure S9**.

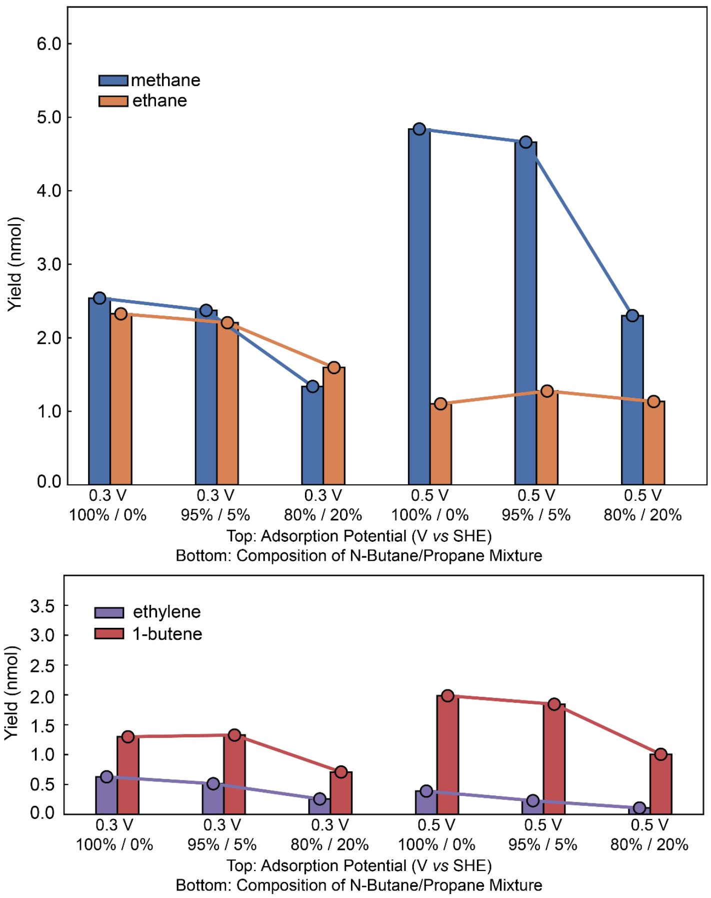

Figure S28. Electrochemical conversion experiments with propane/n-butane mixtures. All listed potentials are referenced to SHE unless otherwise noted. The volumetric ratios of n-butane and propane are labeled in the figure using the format "*x*% / (100 − *x*)%", where *x*% indicates the volumetric percentage of n-butane. Because of the presence of residual propane and n-butane, we focus on the product yields of C_1, C_2 products, and 1-butene. The reported values are the averages of two independent experiments.

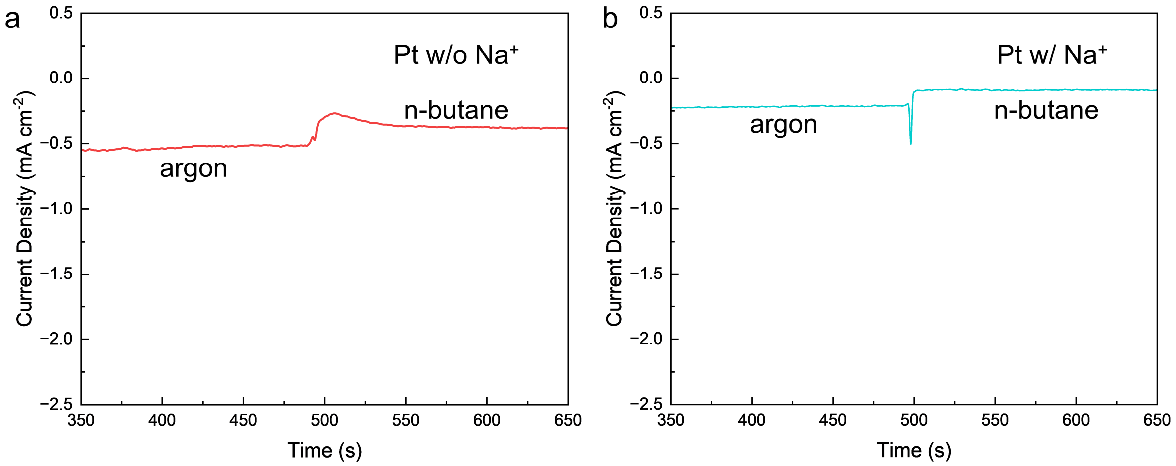

Figure S29 Current measured in different electrolytes during adsorption on the Pt GDE. The electrolyte without Na^+ was 1 M $HClO_4$ and the adsorption potential was 0.3 V *vs* SHE. The electrolyte with Na^+ was 0.001 M $HClO_4$ with 0.1 M $NaClO_4$ and the adsorption potential was 0.3 V *vs* RHE.

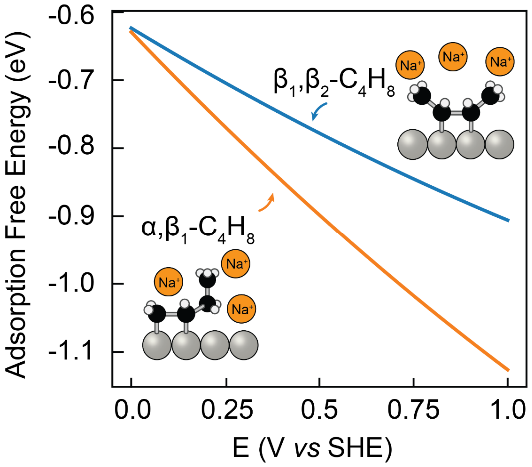

Figure S30. DFT-derived adsorption energy of n-butane fragments (C_4H_8) in the presence of Na^+ cations as a function of the potential. It is worth noting that the position of the Na^+ is for illustrative purposes only.

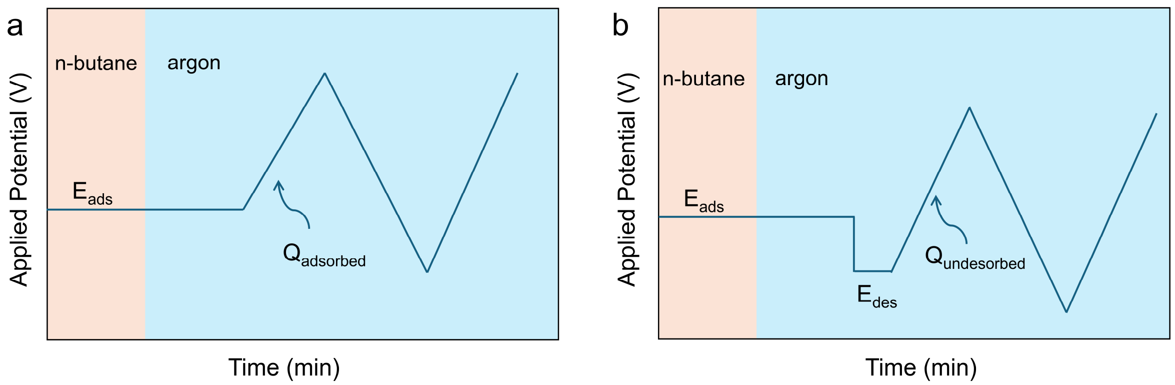

Figure S31. Potential profile of the oxidative stripping experiment. Under n-butane atmosphere, the adsorption potential was held constant for 15 minutes. After switching the gas supply to Ar, the potential was held at the same value for another 15 minutes. Then, oxidative stripping was carried out by cyclic voltammetry at a scan rate of 5 mV s^{-1} **(a)** immediately after adsorption or **(b)** after applying a reductive desorption potential for 5 minutes. The working electrode was a Pt GDE. For the exact adsorption and desorption potentials, please refer to the caption of **Figure S32**.

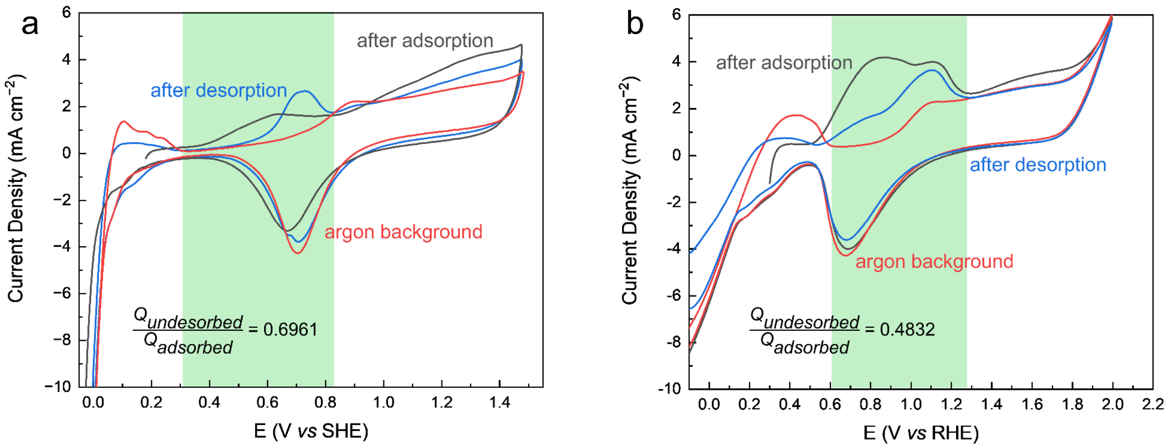

Figure S32. Oxidative stripping results under different conditions. (a) 1 M $HClO_4$ electrolyte without Na^+, adsorption at 0.3 V *vs* SHE, desorption at 0.05 V *vs* SHE. The potential scan range for the cyclic voltammetry was from –0.2 V to 1.5 V *vs* SHE. The scan rate was 50 mV/s. **(b)** 0.001 M $HClO_4$ electrolyte with 0.1 M $NaClO_4$, adsorption at 0.3 V *vs* RHE, desorption at 0.05 V *vs* RHE. The potential scan range for the cyclic voltammetry was from –0.2 V to 2.0 V *vs* RHE. The scan rate was 50 mV/s. Different Pt GDEs were used for tests in different electrolytes. To calculate the charge, we selected the cyclic voltammetry curve under Ar atmosphere in each electrolyte as the respective baseline. The potential range used for integration is indicated by the green shaded area in the figure.

The difference in the CV traces observed before and after reductive desorption depends on whether the electrolyte is dominated by H^+ or Na^+ ions. It can be seen in both cases that the oxidation feature at lower overpotential decreases in intensity after reductive product desorption. However, in presence of Na^+, the difference between the intensity of the low overpotential feature before and after reductive desorption is stronger than in pure acid. This might indicate that the presence of Na^+ promotes the formation of reductively desorbable compounds upon n-butane adsorption, which we indeed observed in our GC measurements (**Figure 4**). This could be due to Na^+-induced changes in adsorption geometry. Interestingly, we also see that in contrast to the purely acidic electrolytes, in presence of Na^+ some compounds appear to remain on the surface after reductive desorption, as indicated by a larger residual oxidative signal. These observations should be the subject of future studies.

5. References

(1) Kovalev, M. K.; Ren, H.; Zakir Muhamad, M.; Ager, J. W.; Lapkin, A. A. Minor Product Polymerization Causes Failure of High-Current CO_2-to-Ethylene Electrolyzers. *ACS Energy Letters* **2022**, *7* (2), 599-601. DOI: 10.1021/acsenergylett.1c02450.

(2) Kresse, G.; Furthmüller, J. Efficiency of Ab-initio Total Energy Calculations for Metals and Semiconductors Using a Plane-wave Basis Set. *Computational Materials Science* **1996**, *6* (1), 15-50. DOI: 10.1016/0927-0256(96)00008-0.

(3) Hammer, B.; Hansen, L. B.; Nørskov, J. K. Improved Adsorption Energetics within Density-functional Theory Using Revised Perdew-Burke-Ernzerhof Functionals. *Physical Review B* **1999**, *59* (11), 7413-7421. DOI: 10.1103/PhysRevB.59.7413.

(4) Grimme, S.; Antony, J.; Ehrlich, S.; Krieg, H. A Consistent and Accurate Ab Initio Parametrization of Density Functional Dispersion Correction (DFT-D) for the 94 Elements H-Pu. *The Journal of Chemical Physics* **2010**, *32* (15). DOI: 10.1063/1.3382344 (acccessed 5/25/2025).

(5) Kresse, G.; Joubert, D. From Ultrasoft Pseudopotentials to the Projector Augmented-wave Method. *Physical Review B* **1999**, *59* (3), 1758-1775. DOI: 10.1103/PhysRevB.59.1758.

(6) Mathew, K.; Kolluru, V. S. C.; Mula, S.; Steinmann, S. N.; Hennig, R. G. Implicit self-consistent Electrolyte Model in Plane-wave Density-functional Theory. *The Journal of Chemical Physics* **2019**, *151* (23). DOI: 10.1063/1.5132354.

(7) Steinmann, S. N.; Michel, C.; Schwiedernoch, R.; Sautet, P. Impacts of Electrode Potentials and Solvents on the Electroreduction of CO_2: A Comparison of Theoretical Approaches. *Physical Chemistry Chemical Physics* **2015**, *17* (21), 13949-13963. DOI: 10.1039/C5CP00946D.

(8) Shen, K.; Cheng, D.; Reyes-Lopez, E.; Jang, J.; Sautet, P.; Morales-Guio, C. G. On the Origin of Carbon Sources in the Electrochemical Upgrade of CO_2 from Carbon Capture Solutions. *Joule* **2023**, *7* (6), 1260-1276. DOI: 10.1016/j.joule.2023.05.010.

(9) Steinmann, S. N.; Sautet, P. Assessing a First-Principles Model of an Electrochemical Interface by Comparison with Experiment. *The Journal of Physical Chemistry C* **2016**, *120* (10), 5619-5623. DOI: 10.1021/acs.jpcc.6b01938.

(10) Cheng, D.; Wei, Z.; Zhang, Z.; Broekmann, P.; Alexandrova, A. N.; Sautet, P. Restructuring and Activation of Cu(111) under Electrocatalytic Reduction Conditions. *Angewandte Chemie International Edition* **2023**, *62* (20), e202218575. DOI: 10.1002/anie.202218575.

(11) Cheng, D.; Nguyen, K.-L. C.; Sumaria, V.; Wei, Z.; Zhang, Z.; Gee, W.; Li, Y.; Morales-Guio, C. G.; Heyde, M.; Roldan Cuenya, B.; et al. Structure Sensitivity and Catalyst Restructuring for CO_2 Electroreduction on Copper. *Nature Communications* **2025**, *16* (1), 4064. DOI: 10.1038/s41467-025-59267-3.

(12) McCrum, I. T.; Hickner, M. A.; Janik, M. J. First-Principles Calculation of Pt Surface Energies in an Electrochemical Environment: Thermodynamic Driving Forces for Surface Faceting and Nanoparticle Reconstruction. *Langmuir* **2017**, *33* (28), 7043-7052. DOI: 10.1021/acs.langmuir.7b01530.

(13) Bhadouria, A.; Heil, J. N.; Parab, D. E.; Greeley, J. P.; Tackett, B. M. Propane Activation on Pt Electrodes at Room Temperature: Quantification of Adsorbate Identity and Coverage. *Angewandte Chemie International Edition* **2025**, *64* (11), e202421613. DOI: 10.1002/anie.202421613.

(14) Li, Y.; Yan, Y.; He, Y.; Du, S. Catalyst Electrodes with PtCu Nanowire Arrays In Situ Grown on Gas Diffusion Layers for Direct Formic Acid Fuel Cells. *ACS Applied Materials & Interfaces* **2022**, *14* (9), 11457-11464. DOI: 10.1021/acsami.1c24010.

(15) Liu, K.; Smith, W. A.; Burdyny, T. Introductory Guide to Assembling and Operating Gas Diffusion Electrodes for Electrochemical CO_2 Reduction. *ACS Energy Lett* **2019**, *4* (3), 639-643. DOI: 10.1021/acsenergylett.9b00137.

(16) Bhadouria, A.; Biswas, A.; Tackett, B. M. Electrocatalytic Transformations of C2 and C3 Hydrocarbons: Bridging from the Past to the Future. *ACS Catalysis* **2025**, *15* (8), 6296-6314. DOI: 10.1021/acscatal.5c00393.
